# A new approach to solving the feature-binding problem in primate vision

**DOI:** 10.1098/rsfs.2018.0021

**Published:** 2018-06-15

**Authors:** James B. Isbister, Akihiro Eguchi, Nasir Ahmad, Juan M. Galeazzi, Mark J. Buckley, Simon Stringer

**Affiliations:** 1 Oxford Centre for Theoretical Neuroscience and Artificial Intelligence, University of Oxford, Oxford OX2 6GG, UK; 2 Oxford Brain and Behaviour Group, Department of Experimental Psychology, University of Oxford, Oxford OX2 6GG, UK

**Keywords:** primate vision, feature-binding problem, spiking neural network, polychronization, binding neuron

## Abstract

We discuss a recently proposed approach to solve the classic feature-binding problem in primate vision that uses neural dynamics known to be present within the visual cortex. Broadly, the feature-binding problem in the visual context concerns not only how a hierarchy of features such as edges and objects within a scene are represented, but also the hierarchical relationships between these features at every spatial scale across the visual field. This is necessary for the visual brain to be able to make sense of its visuospatial world. Solving this problem is an important step towards the development of artificial general intelligence. In neural network simulation studies, it has been found that neurons encoding the binding relations between visual features, known as binding neurons, emerge during visual training when key properties of the visual cortex are incorporated into the models. These biological network properties include (i) bottom-up, lateral and top-down synaptic connections, (ii) spiking neuronal dynamics, (iii) spike timing-dependent plasticity, and (iv) a random distribution of axonal transmission delays (of the order of several milliseconds) in the propagation of spikes between neurons. After training the network on a set of visual stimuli, modelling studies have reported observing the gradual emergence of polychronization through successive layers of the network, in which subpopulations of neurons have learned to emit their spikes in regularly repeating spatio-temporal patterns in response to specific visual stimuli. Such a subpopulation of neurons is known as a polychronous neuronal group (PNG). Some neurons embedded within these PNGs receive convergent inputs from neurons representing lower- and higher-level visual features, and thus appear to encode the hierarchical binding relationship between features. Neural activity with this kind of spatio-temporal structure robustly emerges in the higher network layers even when neurons in the input layer represent visual stimuli with spike timings that are randomized according to a Poisson distribution. The resulting hierarchical representation of visual scenes in such models, including the representation of hierarchical binding relations between lower- and higher-level visual features, is consistent with the hierarchical phenomenology or subjective experience of primate vision and is distinct from approaches interested in segmenting a visual scene into a finite set of objects.

## 1. Introduction

The feature-binding problem concerns how the visual system represents the hierarchical relationships between features (such as edges and objects). For example, at an object level, how does the visual system represent which low-level features belong to a particular object? If two letters T and L are seen together, how does the visual system represent which horizontal and vertical bars are part of which letter?

Moreover, the visual system must represent hierarchical-binding relations across the entire visual field at every spatial scale and level in the hierarchy of visual primitives. Representing the binding relations between visual features is necessary in order for the visual brain to make sense of its visuospatial world. Furthermore, the binding of subfeatures to their parent object would provide rich representations if applied not only to the visual but also to the auditory and behavioural systems of the brain. Consequently, solving this problem would be an important step towards the development of what is commonly termed artificial general intelligence (AGI). This refers to machines that may one day be able to perceive and comprehend their visuospatial environment with a similar semantic richness to the brain, and exploit this semantically rich representation of the world to guide general intelligent behaviour within complex real environments.

**Figure 1.**
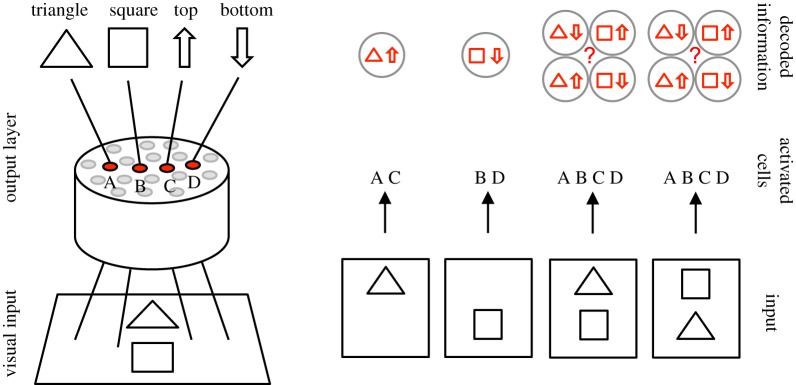
A connectionist example of the feature-binding problem proposed by Rosenblatt [1]. Top row: A neural network receives input from a simple visual scene, in which a triangle or a square can appear in either the top or bottom location. The network has four output neurons A, B, C and D that respond to the following kinds of visual inputs: (A) triangle in either location, (B) square in either location, (C) either object in the top location and (D) either object in the bottom location. Bottom row: The responses of the output neurons to four different visual scenes. It is evident that when a single object is presented, then the combined activity among the output neurons is sufficient to determine the identity of the object and its location. However, when both objects are presented together in different locations, then it is not possible to determine the locations of each of the objects from the responses of the output neurons. Reproduced with permission from Rosenblatt [1]. (Online version in colour.)

One simple example of the feature-binding problem from a connectionist perspective was discussed by Rosenblatt [1] and further elaborated by von der Malsburg [2]. The example is illustrated in figure 1. Consider a neural network with four output neurons A, B, C and D. The first two neurons, A and B, represent the triangle and square, respectively. These neurons have location-invariant responses in that the neurons respond to their preferred objects in both the top and bottom locations. The second pair of output neurons, C and D, represent object location, and respond when any one of the objects is presented in either the top or bottom location, respectively. If the network is presented with a single object, the responses of the output neurons are sufficient to determine the identity of the object and its location. However, when both objects are presented to the network in different positions simultaneously, then all of the output neurons respond and their combined activity is insufficient to determine which object is in which location. This has been called the superposition catastrophe [2]. So how might the visual brain represent which features or objects are in which retinal locations when multiple objects are presented together within a scene?

One approach that has been proposed for solving the feature-binding problem is known as feature integration theory (FIT) [3]. This theory makes the assumption that there is only a single spatial locus of attention within the visual field where features are bound together. This implies that visual tasks requiring feature binding would need to be carried out in a time-consuming serial manner as the visual brain processes the visual field sequentially. However, feature binding would be far more adaptive for an animal if it could be simultaneously performed across the entire visual field in parallel. Moreover, can the feature-binding problem, in which the brain must represent the hierarchical relations between visual features at different spatial scales, really be solved by trying to reduce the size of the spatial region in which it is performed? In fact, an experimental study carried out by Duncan & Humphreys [4] on human participants did not observe a clear dichotomy between serial and parallel modes of visual search. Instead, the search efficiency was related to factors affecting the intrinsic difficulty of the task. For example, the search efficiency decreased as the targets and non-targets became more similar, or if the non-targets became more dissimilar to each other. These experimental observations are inconsistent with the assumption of FIT that visual binding is performed sequentially as a spatial locus of attention shifts across the visual field.

Another mechanism that has been proposed for solving feature binding is synchronization of neuronal firing. Real neurons in the brain communicate with each other by emitting electrical pulses known as action potentials or ‘spikes’. The binding by synchrony hypothesis suggests that the subpopulation of neurons encoding the visual features that are part of the same object will emit their spikes close together in time, but not at the same time as those neurons encoding features associated with different objects [5–7]. In this way, it is suggested that synchronization may be used to segment a visual scene into several discrete object regions. It is important to note that synchronization and oscillations are often interchangeably discussed in the literature with overlapping definitions. In this paper, we use the term synchronization and synchrony to refer to the event when multiple neurons fire spikes effectively simultaneously. Oscillations instead refer to the longer timescale waves of excitatory and inhibitory activity in a network that can be of a width covering tens of milliseconds.

Under the hypothesis of binding by synchrony, simultaneous firing of neurons binds together the visual features that they represent. Attempts to find such a relationship have been unsuccessful [8]. Furthermore, if neural network models incorporate randomized distributions of axonal transmission delays of the order of several milliseconds as found in the brain, then this has the effect of degrading the emergence of synchrony in these simulations. Meanwhile, the question remains: Can decomposing natural scenes into a several-object region really capture the semantic richness of primate vision? Duncan & Humphreys [4] describe the hierarchical nature of primate vision as follows:

A fully hierarchical representation is created by repeating segmentation at different levels of scale. Each structural unit, contained by its own boundary, is further subdivided into parts by the major boundaries within it. Thus, a human body may be subdivided into head, torso, and limbs, and a hand into palm and fingers. Such subdivision serves two purposes. The description of a structural unit at one level of scale (animal, letter, etc.) must depend heavily on the relations between the parts defined within it (as well as on properties such as colour or movement that may be common to the parts). Then, at the next level down, each part becomes a new structural unit to be further described with its own properties, defined among other things by the relations between its own subparts. At the top of the hierarchy may be a structural unit corresponding to the whole input scene, described with a rough set of properties (e.g. division into light sky above and dark ground below).

How might the visual cortex represent such a hierarchy of visual features, as well as the hierarchical binding relations between these features, at every spatial scale and across the entire visual field? Eguchi *et al.* [9] have recently shown how this may be achieved within a biologically realistic hierarchical neural network model of the primate ventral visual system with the following properties.

(1) The model is a ‘spiking’ neural network, in which the timings of the spikes emitted by neurons are explicitly represented.
(2) The synaptic connections are modified during visual training by spike time-dependent plasticity (STDP). Specifically, a synapse is strengthened through long-term potentiation (LTP) if a spike from the presynaptic neuron arrives at the postsynaptic neuron just before the postsynaptic neuron emits a spike. The synapse is weakened through long-term depression (LTD) if the spike from the presynaptic neuron arrives at the postsynaptic neuron just after the postsynaptic neuron has emitted its spike [10,11].
(3) The network architecture incorporates bottom-up, top-down and lateral synaptic connections. This kind of synaptic connectivity is consistent with the primate visual cortex.
(4) There is an axonal transmission delay of a few milliseconds in the time it takes for an action potential or spike to pass from one neuron to another. The axonal transmission delay between each pair of pre- and postsynaptic neurons has a fixed value that does not alter through time. However, different axonal connections have different random transmission delays, which can be anywhere from a few milliseconds to tens of milliseconds.
(5) The network may incorporate multiple synaptic connections between each pair of pre- and postsynaptic neurons, where these connections have different axonal transmission delays. Eguchi *et al.* [9] showed that this allows the STDP to selectively strengthen specific synaptic connections with particular axonal transmission delays.

**Figure 2.**
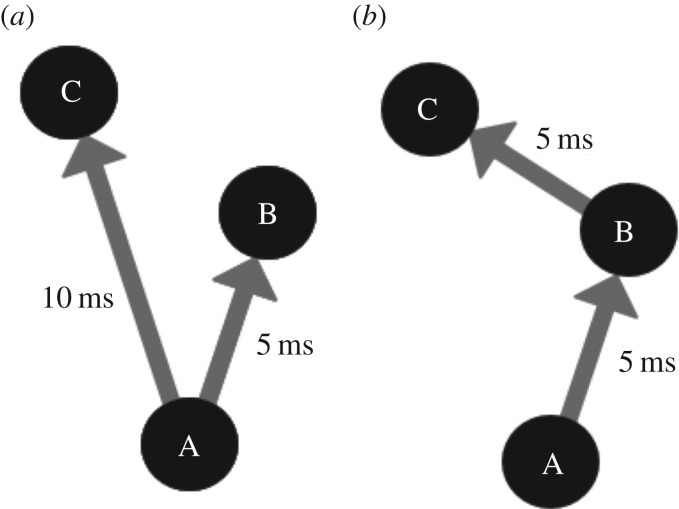
Two minimal example connectivities that could facilitate basic polychronous groups. It is important to note that these are monosynaptic connectivity examples, where only one spike is needed to activate a postsynaptic neuron. While neurons in some areas can be activated by single afferent neurons (some neurons in the lateral geniculate nucleus for example), neurons usually require spikes from multiple neurons to spike. These examples, however, serve the purpose of illustrating the concept of polychronous groups. (*a*) In this example, a spike from neuron A would cause neurons B and C to spike 5 and 10 ms later, respectively. The sequential spike times of A followed by B followed by C are an example of the spatio-temporal pattern of spikes or a polychronous group. (*b*) The connectivity of this example would cause the same spatio-temporal pattern of spikes as example (*a*) but instead neuron B would cause neuron C to fire. (*a*) and (*b*) are both examples of polychronous groups caused by different underlying connectivities. Copyright © 2018 American Psychological Association. Reproduced [or Adapted] with permission. The official citation that should be used in referencing this material is [9]. No further reproduction or distribution is permitted without written permission from the American Psychological Association.

Using a neural network model with the above architectural components, Eguchi *et al.* [9] reported that training the model on visual stimuli led to the emergence of repeating spatio-temporal patterns of spikes in the higher layers of the network. A subpopulation of such neurons that emit their spikes in a regularly repeating spatio-temporal chain is referred to as a polychronous neuronal group (PNG). Figure 2 illustrates two examples of basic network connectivities, which could underlie basic polychronous groups. The phenomenon of network responses with spatio-temporal patterns of neural activity is known as polychronization [12]. This is in contrast with synchronization, in which the spikes of subpopulations of neurons are clustered very close together in time (synchronized). A key factor in pushing the network from synchronous to polychronous activity is the incorporation of axonal transmission delays, which forces the neurons to spike at different times relative to other neurons. The original study also reported that these PNGs responded selectively to particular visual stimuli on which the network had been trained. These stimulus-specific PNGs were found to emerge even though the input neurons representing the visual stimuli had entirely randomized spike times set according to a Poisson distribution.

Neurophysiological evidence has emerged for the existence of stimulus-specific and reliable patterns in spike timing. Havenith *et al*. [13] showed the existence of high levels of stimulus-specific information in the timing of action potentials. Notably, the timing of these spikes was reliable relative to the peak of the underlying neuron population oscillation (beta/low gamma) and not relative to stimulus onset. The function and relevance of underlying population oscillations (transient or otherwise) is intriguing and the evidence strong for a functional role in activity gating, information propagation and more (for a review see [14]), though we leave an exploration of the theoretical benefits of such oscillations to a future study. Nonetheless, this observed timing was in the context of a range of visual stimuli (drifting gratings with motion in differing angles) and recorded in the primary visual cortex [13]. This evidence lends support to the idea that spike times are an informative property of neuronal responses. Evidence has also been found for precise spike timing with respect to stimulus onset in the early visual system [15–17]. The interaction between cortical oscillations and of precise spike timing relative to stimulus onset may also be of importance.

A key conceptual development by Eguchi *et al.* [9] was to propose that embedded within these PNGs exist neurons, called binding neurons, that learn to represent the hierarchical binding relationships between lower- and higher-level visual features. The simulations performed by these authors demonstrated that such binding neurons develop automatically and robustly within the emergent PNGs during visual training. Moreover, in theory, these kinds of neurons will learn to represent the hierarchical binding relations between visual features at every spatial scale and across the entire visual field. Thus, models exploiting polychronization may enable a richer representation of a visual scene than that permitted by either FIT or feature binding by synchronization. In particular, the hierarchical representations of visual scenes that emerge in the models investigated by Eguchi *et al.* [9], including the representation of hierarchical binding relations between lower- and higher-level visual features, are consistent with the hierarchical subjective experience of primate vision as described by Duncan & Humphreys [4].

A further hypothesis of the original study was that information about visual features at every spatial scale, including the binding relations between these features, would be projected upwards to the higher layers of the network, where such fine-grained spatial information would be available for readout by later brain systems to guide behaviour. The authors referred to this as a holographic principle. Such a mechanism could be important in the primate brain if subsequent brain regions that are responsible for decision-making and behaviour only receive connections from the higher stages of the visual system. Consistent with this, a neurophysiology study carried out by Rainer *et al.* [18] showed that information about the location of a target object was encoded in the responses of neurons in the prefrontal cortex (PFC). The simulations carried out by Eguchi *et al.* [9] also provided evidence for this hypothesized upwards projection of visual information.

In this paper, we review the theory of hierarchical feature binding proposed by Eguchi *et al.* [9], their spiking neural network model and simulation results. As discussed above, the simulation studies carried out by these authors reported the emergence of stimulus-specific spatio-temporal patterns of spikes (PNGs) within the higher network layers, which are repeated across different presentations of the same stimulus, even when the spike timings of the stimulus representations in the input layer were randomized. These authors investigated the emergence of both large-scale PNGs consisting of many neurons, as well as spike-pair PNGs consisting of just two neurons that carried high levels of stimulus-specific information. However, there is a potential issue with the latter results. Consider two neurons that respond selectively to a particular preferred stimulus with high firing rates, but do not respond to any other stimuli. In this case, it may be possible to find what appear to be spike-pair PNGs, i.e. particular interspike intervals that are repeated across a large proportion of presentations of the preferred stimulus, even though the times of spikes emitted by the two neurons may in fact be random. In the light of this possibility, in §4.1 we also present some new simulation results taking a closer look at the gradual emergence of spatio-temporal structure (polychronization) as signals progress through a hierarchy of network layers.

## 2. Theory

### 2.1. The emergence of polychronization within a biological spiking neural network model of the primate visual system

Eguchi *et al.* [9] hypothesized that the kind of spiking neural network architecture with properties (i)–(iv) described above, especially including randomized distributions of axonal transmission delays, would develop regularly repeating spatio-temporal patterns of spiking activity in the higher network layers after training on a set of visual stimuli—i.e. polychronization [12]. This hypothesis was originally inspired by the modelling study of Diesmann *et al.* [19]. They showed that a hierarchical spiking neural network consisting of series of successive layers could lead to the emergence of synchronous activity in the higher layers even when the spikes in the input layer were widely dispersed (i.e. unstructured) in time. This is an example of the development of a synfire chain. Synfire chains were originally proposed by Abeles [20] as networks within which such synchronous activity could propagate. They are defined as a hierarchical series of pools (or layers) of neurons which when a given layer fires, in a sufficiently synchronous manner, the resulting volley of spikes propagate on from each pool to the next, causing each pool of neurons to fire synchronously one after another in sequence. The conditions for the stability of synfire chains have been explored and characterized, and a major requirement within these networks is the existence of single-valued synaptic transmission delays [19,21]. The reason this work was impactful was that feature binding was posited at the time to be linked to synchronized neuronal activity, whereby the spikes emitted by neurons representing visual features that are part of the same object would be clustered very closely together in time. The simulations of Diesmann *et al.* [19] showed how such synchronized activity could emerge naturally within a biological spiking neural network. However, in order for synchrony to emerge in their simulations, the model assumed either no axonal delays or axonal delays all of the same fixed length. Bienenstock [22] proposed that it might be possible to relax this constraint by allowing the incorporation of non-uniform axonal transmission delays as actually found in the brain. These authors hypothesized that synchronous waves could still emerge if pairs of given neurons in the network were connected by multiple polysynaptic pathways with the same overall length. This was referred to as a synfire braid. Nevertheless, Bienenstock [22] was still concerned with the emergence of synchronized activity to solve feature binding. As discussed above, our view was that synchrony could not offer a solution to the binding problem that accorded with the rich hierarchical phenomenology of primate vision [4]. In this paper, we shall be discussing the alternative notion of polychronization proposed by Izhikevich [12], and its potential role in solving feature binding. Although synfire chains/braids and polychronization both involve spatio-temporal patterns of spiking activity, the latter is quite distinct from the former. Specifically, polychronization is far less constrained than synfire chains/braids in that it does not assume that subpopulations of neurons have to emit their spikes in a synchronized manner. We have found that this greater freedom can lead to the emergence of representations of the hierarchical binding relations between lower- and higher-level features.

Building closely on the work of Diesmann *et al.* [19], it was hypothesized by Eguchi *et al.* [9] that including randomized distributions of axonal transmission delays, e.g. spread uniformly in the range 0–10 ms, into such hierarchical spiking network models would force neurons to emit their spikes separated in time, thereby creating spatio-temporal spike sequences (PNGs). Moreover, as Diesmann *et al.* [19] showed how synchronous neural activity could gradually emerge through successive layers even when there was no such temporal structure among spikes in the input layer, Eguchi *et al.* [9] hypothesized that input patterns with randomized spike times could lead to the emergence of polychronous activity in the higher layers of networks incorporating randomized distributions of axonal delays. In the simulation study reported in Eguchi *et al.* [9], the spike patterns representing the stimuli in the input layer had no regular temporal structure, except that the average firing rates of the input neurons were set in accordance with the outputs of Gabor filters that simulated the responses of simple cells in visual area V1. Eguchi *et al.* [9] also hypothesized that training the network on visual stimuli using STDP to modify the synaptic connections would enhance the emergence of PNGs in the higher network layers, where individual PNGs would learn to respond to a particular preferred visual stimulus. The study reported that these predictions were confirmed in their simulations. Moreover, these authors reported that many more stimulus-specific PNGs emerged in the highest (output) layer than individual neurons tuned to specific visual stimuli. This strongly hints at such PNGs playing an important role in stimulus representations in the brain. This is supported by experimental observations from multi-unit recording studies in monkeys, which have reported the existence of such spatio-temporal spike patterns in the primate cortex in response to the presentation of visual stimuli [23,24].

Given the reported emergence of stimulus-specific polychronous activity in the spiking network simulations performed by Eguchi *et al.* [9], and the observed presence of these kinds of PNGs in the primate brain, what role might such polychronous activity play in solving the feature-binding problem in a way that reflects the hierarchical subjective experience of primate vision?

### 2.2. How the emergence of polychronization may offer an approach to solving feature binding in primate vision

Eguchi *et al.* [9] proposed that the emergence of polychronous activity within a hierarchical spiking neural network may provide an understanding of how the primate brain solves the feature-binding problem. To address the hierarchical phenomenology of primate vision, as described by Duncan & Humphreys [4], consider a couple of higher-level features or stimuli such as the alphabetical letters T and L, each of which may be located anywhere on the retina. The letters T and L both comprise a horizontal bar and a vertical bar, which are the lower-level features. If the letters T and L are presented together at some random locations on the retina, how might the visual cortex represent which horizontal and vertical bars (lower-level features) are part of which letters (higher-level features or stimuli)? The ability to represent such hierarchical binding relations between lower- and higher-level features is fundamental to the ability of the visual brain to produce an integrated representation of a visual scene, and consequently make sense of its visuospatial world.

Consider training a spiking network with properties (i)–(iv) described above on the letter T presented everywhere across the retina. Eguchi *et al.* [9] hypothesized that this would lead to the emergence of a T-specific PNG which is activated regardless of the location of the T on the retina. Within this T-specific PNG, it was hypothesized that binding neurons could exist which encode the hierarchical binding relations between lower- and higher-level features. Specifically, such binding neurons were posited to fire as part of the PNG if, and only if, a neuron or subset of neurons representing a lower-level feature, such as a horizontal bar at a particular retinal location, was participating in driving the neuron or subset of neurons representing a higher-level feature, such as the letter T. In this case, the binding neurons would carry measurable information that the lower-level feature was part of the higher-level feature or stimulus.

**Figure 3.**
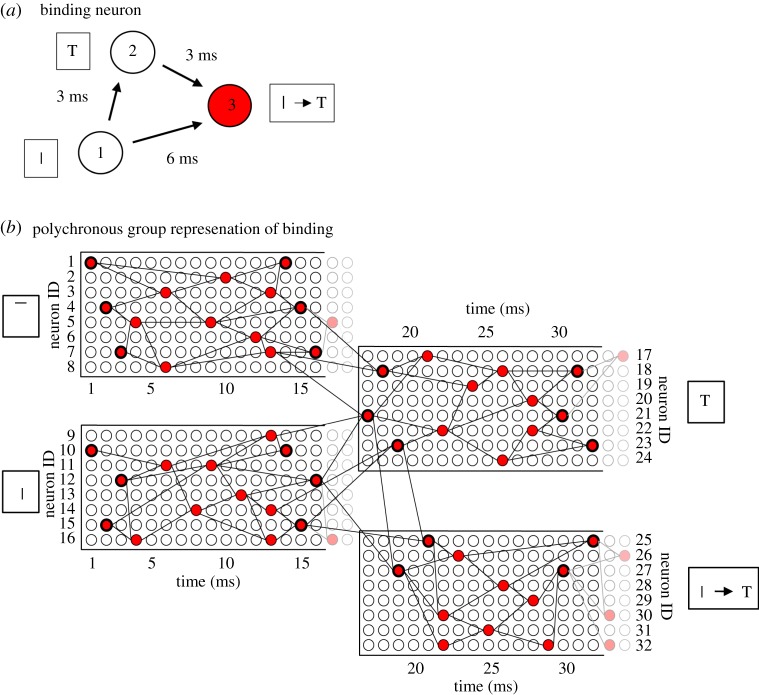
(*a*) Simplest example of a binding neuron. There are three linked neurons forming a three-neuron binding circuit with non-zero axonal transmission delays between the neurons. Neuron 1 is in a lower layer and represents a lower-level feature such as a vertical bar, neuron 2 is in a higher layer and represents a higher-level feature such as a letter T, and neuron 3 is the binding neuron whose activity represents that the lower-level feature is part of the higher-level feature. In an ideal network, the synapses would have fast synaptic time constants so that multiple presynaptic spikes need to arrive simultaneously at a postsynaptic neuron in order for the neuron to reach its voltage threshold and fire. By examining the magnitudes of the axonal delays shown in the figure, it can be seen that the spikes from neurons 1 and 2 will arrive at neuron 3 simultaneously and cause it to fire at the correct time *if and only if* neuron 1 is participating in driving neuron 2. Consequently, the activity of binding neuron 3 will effectively represent the fact that the lower-level feature (the vertical bar) represented by neuron 1 is part of the higher-level feature (the letter T) represented by neuron 2. (*b*) PNG representations of features and binding relations. Rather than by individual neurons, it is likely that lower- and higher-level visual features such as the vertical bar and letter T, respectively, are represented by their own PNGs. Moreover, the hierarchical binding relationship between the lower- and higher-level features is likely to be also represented by its own PNG. In this case, the simplified three-neuron binding circuit shown in (*a*) would be part of the more complex neural response set-up illustrated in (*b*). Reproduced with permission from Eguchi *et al.* [9]. Copyright © 2018 American Psychological Association. Reproduced [or Adapted] with permission. The official citation that should be used in referencing this material is [9]. No further reproduction or distribution is permitted without written permission from the American Psychological Association. (Online version in colour.)

The simplest example of how such binding neurons might operate is shown in figure 3*a*. There are three neurons forming a binding circuit. Neuron 1 in a lower layer represents the low-level feature; neuron 2 in the higher layer represents the high-level feature; and neuron 3 is a binding neuron that encodes the hierarchical binding relation between the low- and high-level features. The connections between the neurons have axonal transmission delays associated with them, where Δ_(*i*,*j*)_ denotes the delay from presynaptic neuron *j* to postsynaptic neuron *i*. Given the existence of the axonal transmission delays shown in figure 3*a*, it is evident that neuron 1 will be participating in driving neuron 2 only if a spike emitted by neuron 2 occurs approximately Δ_(2,1)_ after a spike emitted by neuron 1. Moreover, if the three axonal delays shown in figure 3*a* have the relationship2.1
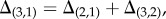
then the spikes emitted by neurons 1 and 2 will arrive at the binding neuron 3 if and only if neuron 1 (representing the low-level feature) is participating in driving neuron 2 (representing the high-level feature). This is of critical importance because it is assumed that all neurons have relatively fast synaptic time constants, as well as synaptic weights appropriately scaled to the synaptic time constant, so that postsynaptic neurons only fire when presynaptic spikes arrive simultaneously. In this case, binding neuron 3 may fire if and only if neuron 1 is participating in driving neuron 2. In other words, the binding neuron 3 may fire if and only if the low-level feature encoded by neuron 1 is part of the high-level feature or stimulus encoded by neuron 2. In this case, the binding neuron 3 will carry measurable information about the hierarchical binding relationship between the lower- and higher-level visual features.

Eguchi *et al.* [9] demonstrated in simulations that such three-neuron binding circuits, with the relationship between the axonal delays shown in equation (2.1), do indeed develop when the network is trained on a set of visual stimuli using STDP to modify the synaptic connections. In particular, in such binding circuits, the low-level feature neuron 1 was in a lower network layer and encoded a relatively simple visual feature, while the high-level feature neuron 2 was in a higher network layer and encoded a more complex visual feature that appeared to incorporate the simple feature encoded by neuron 1. Moreover, the three neurons did indeed fire in the way hypothesized when current was artificially injected into the network. That is, high-level feature neuron 2 emitted a spike Δ_(2,1)_ after low-level feature neuron 1, and the binding neuron 3 emitted a spike Δ_(3,2)_ after high-level feature neuron 2. Some of the simulation results carried out by Eguchi *et al.* [9] are shown below. An important note is that the synaptic time constants for the excitatory-to-excitatory connections actually used in the original study were, in fact, set to the relatively slow value of 150 ms as this same network was later used for a task requiring the development of translation invariance using trace learning (as applied by Evans & Stringer [25]). A faster synaptic time constant would ensure that the effect of an incoming spike would decay rapidly, and, therefore, the simultaneous arrival of multiple incoming spikes would be required for activation of a binding neuron. Consequently, we would expect that a faster synaptic time constant would further encourage the neurons to act as coincidence detectors, and such a set-up could result in the emergence of many more binding neurons. It should also be noted that these authors trained and tested their spiking network on the rather impoverished stimulus set shown in §4.2.1. In particular, they did not test the firing responses of such three-neuron binding circuits on a large set of realistic visual objects translating across different retinal locations, with multiple objects being presented together simultaneously during testing. These kinds of more realistic simulation are needed to enable a proper test of whether such binding neurons consistently fire if and only if the low-level feature neuron 1 is participating in firing the high-level feature neuron 2.

Nevertheless, to reiterate, according to the overarching hypothesis the binding neuron should fire ‘if and only if’ the neuron encoding the lower-level feature is participating in driving the neuron encoding the higher-level feature. Only in this condition will the binding neuron represent the hierarchical binding relationship between the two features. This emergent firing property of the binding neuron relies on the spiking dynamics of the model, and, in particular, the operation of the binding neuron as a ‘coincidence detector’ that requires multiple spikes to arrive independently to fire. In a more standard rate-coded neural network, which does not explicitly simulate the timings of action potentials or spikes, such a binding mechanism would not be possible. The spiking network architecture and dynamics described above enable the binding neuron to selectively not respond when the neurons encoding the lower- and higher-level features are co-active in a non-causal fashion. For example, if the letters T and L are presented together, then the neuron representing the vertical bar of the T and the neuron representing the letter L will both be active, but the former will not participate in driving the latter. In this case, the binding neuron linking the vertical bar of the T to the letter L will not fire because the afferent spikes do not arrive simultaneously.

However, in reality, it is more likely that the lower- and higher-level visual stimuli would be represented by PNGs in their own right. Similarly, the hierarchical binding relationship between these features could be represented also by a PNG. Such an arrangement is illustrated in figure 3*b*.

A key advantage of the general polychronous binding mechanism (illustrated in figure 3) over FIT is that the former binding mechanism, which relies on the emergence of polychronization within a biological spiking neural network, could operate at every spatial scale and over the entire retinal field of vision. In this way, the proposed binding mechanism could provide a rich hierarchical representation of the visual features across a scene at every spatial scale, as well as the hierarchical binding relations between these features, in a manner consistent with the hierarchical phenomenology of primate vision described by Duncan & Humphreys [4]. This also differs from the binding-by-synchrony hypothesis, in which the features can only be segmented into a small number of separate groups.

The proposed solution to the binding problem could be considered a form a combination coding, in that binding neurons learn to respond to the combination of a low and high level with high specificity. Overall, however, the representation of a high-level feature and its comprising binding relations form a distributed code across binding neurons. In the context of hardwired versus on-demand binding, the proposed solution is currently a form of hard-wired binding, representing binding relations that have previously been exposed to the network. How the mechanism generalizes to represent novel binding relations is yet to be explored.

### 2.3. Bottom-up projection of visual information about low-level elemental features to higher network layers

Eguchi *et al.* [9] also hypothesized that the kind of mechanisms described above could also lead to visual information at smaller spatial scales being projected up to the higher layers of the network, which they called the *holographic principle*. The traditional view of processing in the primate ventral visual pathway is that the scale and complexity of visual features that are represented grow as one ascends the hierarchy of processing stages or layers. For example, it is widely thought that neurons in early cortical visual areas such as V1 and V2 represent local oriented bars and edges, while neurons in higher cortical areas such as the anterior inferior temporal cortex (TE) encode whole objects and faces. However, surely subsequent brain areas such as the PFC that are responsible for decision-making and behaviour must be guided by visuospatial representations at every spatial scale? If such behavioural brain areas only receive inputs from the later stages of the visual system, then there must be some way in which information about visual features at every spatial scale, including the binding relations between these features, is projected up to the higher visual layers for readout by later behavioural brain systems. Neurophysiology experiments on primates seem to support this proposal.

For example, Rainer *et al.* [18] recorded the responses of neurons in the PFC, a brain area that receives inputs from the higher cortical visual stages and which is involved in decision-making. It was found that individual PFC neurons responded differentially depending on the location of the target stimulus, which is analogous to different sets of low-level features driving the target representation. This indicates that PFC is encoding the spatial configuration of visual objects rather than just the identity of the whole objects themselves.

**Figure 4.**
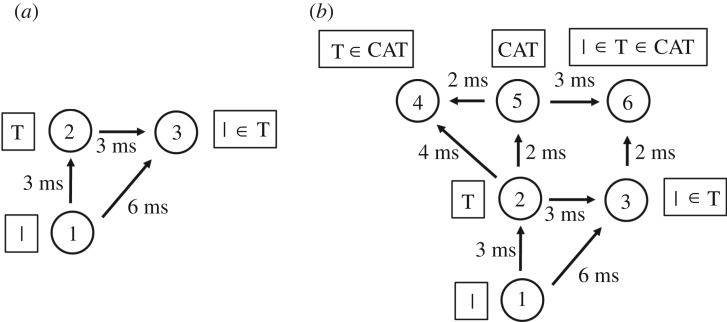
The general binding mechanism illustrated in figure 3 permits information about low-level visual features to be projected up to the higher layers of the network, where such information may be used by later brain systems to guide decision-making and behaviour. (*a*) A three-neuron binding circuit which is similar to that shown in figure 3*a*. However, now the binding neuron 3 is located in the higher layer along with neuron 2 representing the higher-level feature. In this case, the binding neuron 3 in the higher layer represents the presence of the lower-level feature (e.g. vertical bar) represented by neuron 1, as well as the fact that the lower-level feature is part of the higher-level feature (e.g. letter T) represented by neuron 2. Thus, information about the presence of the lower-level feature, and the fact that it is part of the higher-level feature, has been projected up to the higher network layer. (*b*) This process could continue up through successive network layers. Here neuron 6 is a form of binding neuron that receives inputs from neurons 3 and 5, and represents the fact that the lower-level feature (e.g. vertical bar) is part of the higher-level feature (e.g. letter T), which in turn is part of the highest level feature (e.g. word CAT). Consequently, information about the lowest level feature is projected up to the highest network layer. Reproduced with permission from Eguchi *et al.* [9]. Copyright © 2018 American Psychological Association. Reproduced [or Adapted] with permission. The official citation that should be used in referencing this material is [9]. No further reproduction or distribution is permitted without written permission from the American Psychological Association.

A very simple mechanism that can lead to information about a lower-level visual feature, including its hierarchical binding relationship with a higher-level visual feature, being projected up to a higher network layer is illustrated in figure 4*a*. This is similar to the network architecture shown in figure 3*a*. However, the binding neuron 3, which represents that the lower-level feature (such as a vertical bar) is part of the higher-level feature (such as the letter T), is now located in the higher layer along with neuron 2 encoding the higher-level feature. In this situation, information about the lower-level feature, and its hierarchical binding relationship with the higher-level feature, has now been projected up to the higher layer. In fact, Eguchi *et al.* [9] found that a large proportion of the three-neuron binding circuits that they developed in their simulations were of this general form, with the binding neuron situated in the higher layer.

This kind of upward projection of visual information about lower-level visual features, and their hierarchical binding relations, may operate at every stage of processing in the visual hierarchy, and operate simultaneously across the visual field. Indeed, figure 4*b* shows how the general process may be repeated through successive network layers. This figure considers a hierarchy of three visual features: the vertical bar is part of a letter T, which in turn is part of the word CAT. The vertical bar is represented by neuron 1 in the lowest layer, the letter T is represented by neuron 2 in the middle layer, and the word CAT is represented by neuron 5 in the highest layer. A hierarchy of such stimulus representations could develop through competitive learning operating in successive network layers during visual training on written text. In this example, binding neuron 3 (representing that the vertical bar is part of the letter T) is situated in the middle layer. Next, neuron 6 in the highest network layer receives combined inputs from binding neuron 3 and neuron 5. Consequently, neuron 6 will represent that the lowest level feature (the vertical bar) is part of the higher feature (the letter T), which in turn is part of the highest level feature (the word CAT). In this way, information about visual features at every spatial scale (vertical bar, letter T and word CAT), including the hierarchical binding relations between all of these features, may be projected up to the highest network layer for readout by later behavioural brain systems. However, it should be noted that, in order for binding neuron 6 to fire, the highest level feature (e.g. the word CAT) must be presented to the network; neuron 6 will not fire to the presence of the lowest level feature (e.g. the vertical bar) alone. This theory is therefore consistent with the experimental observation that neurons in the higher stages of the ventral visual pathway tend to be preferentially activated by more complex visual forms than neurons in early cortical areas such as V1 and V2 which can respond to relatively simple oriented bars and edges.

As discussed above, the traditional view of hierarchical processing in the ventral visual pathway is that successive stages of processing learn to represent stimuli of increasing scale and complexity. Specifically, neurons in lower cortical visual areas such as V1 and V2 encoding lower-level visual features such as oriented bars and edges typically have small receptive fields of about 1° or 2°. As one ascends the visual hierarchy, stimulus representations tend to become more transform invariant. That is, a neuron in a higher stage of processing that represents an object or face may respond to its preferred stimulus across different retinal locations, or viewpoints or distances (in effect scales) [26–28]. Given such transform invariance, how do the higher visual areas represent the exact location of a complex stimulus?

The upward projection of information about visual features at every spatial scale, including the binding relations between these features, provides a mechanism by which the higher stages of the visual cortex may localize visual features, and, consequently, the objects comprised of those features in (retinal) space. Moreover, as described by Duncan & Humphreys [4], the primate brain produces a rich hierarchical representation of the visual world, in which we see a hierarchy of visual features as well as the binding relations between these features. In particular, we are aware of where all of the features at every spatial scale are located in space.

The holographic upward projection of visual information described above provides a mechanism by which the higher stages of processing in the visual brain may represent such fine-grained spatial information about a visual scene. Then, when this kind of low-level visuospatial information is projected upwards to higher layers, including information about the binding relations between the lower- and higher-level visual features, this will enable the higher visual layers to represent where (parts of) complex stimuli such as objects and faces are located in space.

### 2.4. Binding neuron activation through local increases in spike density

**Figure 5.**
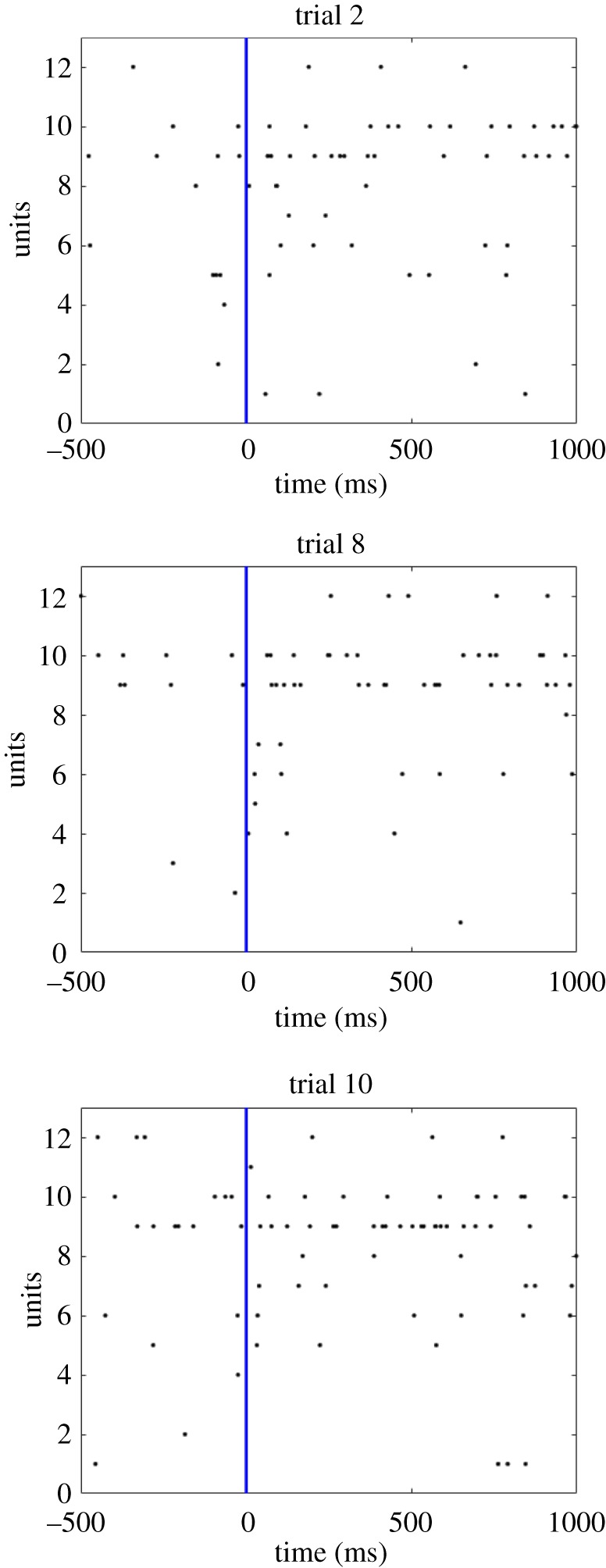
Neurophysiological evidence from single unit recording carried out in monkey prefrontal cortex (PFC) using chronically implanted multi-electrode arrays. This figure presents three spike raster plots recorded from the PFC of an awake behaving monkey as the animal looked at a visual stimulus. That is, each row shows the series of spikes emitted by a different individual unit through 1000 ms as the monkey viewed the stimulus. It can be seen that each spike raster plot shows fluctuations in the local spike density through time, with some localized clusters of spikes. We hypothesize that these localized variations in spike density reflect underlying polychronous activity within this brain region and other reciprocally connected areas. (Online version in colour.)

The original hypothesis of Eguchi *et al.* [9] was that polychronous activity depended on precise spatio-temporal patterns of individual spikes emitted at specific times with millisecond precision. For example, in the binding circuit illustrated in figure 3*a*, it was assumed that neuron 1 would emit a single spike at time zero, then neuron 2 would emit a single spike 3 ms after neuron 1 and then binding neuron 3 would emit a single spike 3 ms after neuron 2. However, we now propose that the binding mechanism could still operate in a somewhat similar manner but instead use local increases in spike density at appropriate moments in time. That is, instead of neuron 2 emitting a single spike exactly 3 ms after neuron 1, the kind of binding mechanism illustrated in figure 3*a* could still operate even if neuron 2 simply displays an increase in the number of spikes emitted around that time, i.e. a temporally localized increase in the spike rate. Examples of how this might look are shown in figure 5, which shows a number of spike raster plots recorded from the PFC of an awake behaving monkey as the animal was presented with a visual stimulus. It is evident that each spike raster plot shows fluctuations in the local spike density through time, with some localized clusters of spikes. This kind of neuronal behaviour, in which there appears to be some kind of regular underlying temporal variation in spike rate, is quite typical in visually responsive neurons in monkey cortex. We hypothesize that these localized variations in spike density through time reflect underlying spatio-temporal activity patterns across subpopulations of neurons, which include neurons carrying information about the (hierarchical) binding relations between visual features. This broader concept, based on local variations in spike density, represents a generalization of the original notion of polychronization in which neurons had to emit single spikes at particular times. However, even with this more flexible form of polychronization, the binding mechanisms illustrated in figure 3*a* should still operate in a similar manner as long as the increases in neuronal spike rate are sufficiently temporally localized. That is, neuron 2 displays a localized increase in spike rate around 3 ms (approx. 2–4 ms) after a localized increase in the spike rate of neuron 1, and binding neuron 3 displays a localized increase in spike rate around 3 ms (approx. 2–4 ms) after a localized increase in the spike rate of neuron 2. We, therefore, suggest that the temporal structure displayed in the spike rasters shown in figure 5 reflects this more generalized form of polychronous activity within the PFC and other reciprocally connected brain areas. In future work, we will use multi-unit recording techniques in monkeys, in which the spiking activity of many neurons is recorded simultaneously, to look for the existence of neurons using this more general form of polychronization to carry measurable information about the (hierarchical) binding relations between visual features.

## 3. Neural network model and analysis of network performance

In this section, we describe the original neural network model and performance analyses employed in the simulation study by Eguchi *et al.* [9]. Then, in §4.1, we present some novel simulation results investigating the emergence of polychronization through successive network layers using a simplified version of this model. Finally, in §4.2, we review some of the original simulation results of Eguchi *et al.* [9] showing the emergence of feature-binding representations within PNGs.

### 3.1. Neural network model

#### 3.1.1. Network architecture

**Figure 6.**
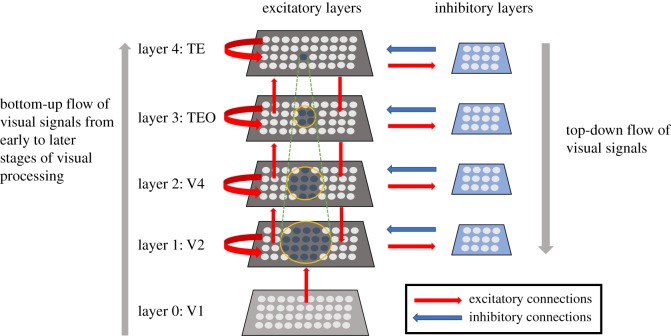
The four-layer neural network model of the primate ventral visual pathway investigated by Eguchi *et al.* [9]. The network architecture consists of a hierarchy of four layers of neurons 1–4 that correspond to cortical visual areas V2, V4, posterior inferior temporal cortex (TEO) and anterior inferior temporal cortex (TE). Within each of these four network layers, there are subpopulations of interconnected excitatory and inhibitory neurons. Layer 0 contains a layer of excitatory neurons, whose firing rates reflect the outputs of Gabor filters that mimic the responses of bar/edge-detecting V1 simple cells after convolution with the visual input image. Although the firing rates of the layer 0 neurons are set according to the outputs of the Gabor filters, their actual spike times are randomized according to a Poisson distribution. Thus, there is no spatio-temporal structure imposed on the spiking activity of the input layer; this has to emerge gradually as visual signals propagate through the hierarchy of higher layers 1–4. Layer 0 neurons have purely bottom-up (feedforward) connections to layer 1. Each of the following layers 1–4 consists of 64 × 64 = 4096 excitatory neurons and 32 × 32 = 1024 inhibitory neurons. The excitatory plastic (modifiable) synaptic connections (shown in red) in the model include bottom-up (feedforward) and top-down (feedback) connections between excitatory neurons in successive layers, and lateral connections between excitatory neurons within the same layer (shown by the curved red arrows). Within each layer, the subpopulation of excitatory neurons send non-modifiable projections to the subpopulation of inhibitory neurons, which in turn send non-modifiable connections back to the excitatory neurons. The inhibitory interneurons effectively implement lateral competition between the subpopulation of excitatory neurons within a layer.

The neural network model investigated by Eguchi *et al.* [9] is shown in figure 6. It simulates successive stages of processing within the primate ventral visual pathway. Specifically, it consists of four hierarchical layers of neurons that correspond to cortical visual areas V2, V4, posterior inferior temporal cortex (TEO) and anterior inferior temporal cortex (TE). Within each network layer, there are subpopulations of interconnected excitatory and inhibitory neurons. There are plastic (modifiable) bottom-up (feedforward) and top-down (feedback) synaptic connections between excitatory neurons in successive layers, as well as plastic lateral synapses between excitatory neurons within each layer. The inhibitory neurons within each layer have non-plastic connections to and from the excitatory neurons. The inhibitory interneurons effectively implement lateral competition between the excitatory neurons within a layer. This supports competitive learning among the excitatory neurons within each layer, whereby individual excitatory neurons learn to respond to particular visual features or stimuli presented during training. There were 64 × 64 = 4096 excitatory neurons and 32 × 32 = 1024 inhibitory neurons within each layer. The excitatory connectivity between layers was topographical, with excitatory neurons in each layer receiving connections from excitatory neurons within a corresponding local region of the lower or higher layer. Table 1*a* shows the different numbers of afferent connections onto each postsynaptic neuron, as well as the fan-in radius of these connections, for the different types of excitatory–excitatory, excitatory–inhibitory and inhibitory–excitatory connections between and within the four layers of the network.

**Table 1.** Model parameters. Most integrate and fire parameters were taken from Troyer *et al.* [30] (derived originally from McCormick *et al.* [31]) as indicated by §. Plasticity parameters (denoted by †) are taken from Perrinet *et al.* [29]. Parameters marked with * were tuned for the reported simulations.

layer	1	2	3	4
(*a*) *network parameters*				
number of excit. neurons within each layer	64 × 64	64 × 64	64 × 64	64 × 64
number of inhib. neurons within each layer	32 × 32	32 × 32	32 × 32	32 × 32
number of feedforward (FF) afferent excit. connections per excit. neuron (EfE)	30	100	100	100
fan-in radius for FF afferent excit. connections to each excit. neuron (EfE)	1.0	8.0	12.0	16.0
number of feedback (FB) afferent excit. connections per excit. neuron (EbE)	{0,10}	{0,10}	{0,10}	—
fan-in radius for FB afferent excit. connections to each excit. neuron (EbE)	8.0	8.0	8.0	—
number of lateral (LAT) afferent excit. connections per excit. neuron (ElE)	{0,10}	{0,10}	{0,10}	{0,10}
fan-in radius for LAT afferent excit. connections to each excit. neuron (ElE)	4.0	4.0	4.0	4.0
number of LAT afferent excit. connections per inhib. neuron (ElI)	30	30	30	30
fan-in radius for LAT afferent excit. connections to each inhib. neuron (ElI)	1.0	1.0	1.0	1.0
number of LAT afferent inhib. connections per excit. neuron (IlE)	30	30	30	30
fan-in radius for LAT afferent inhib. connections to each excit. neuron (IlE)	8.0	8.0	8.0	8.0
(*b*) *parameters for Gabor filtering of visual images*				
phase shift (*ψ*)	0, *π*			
wavelength (*λ*)	2			
orientation (*θ*)	0, *π*/4, *π*/2, 3*π*/4			
spatial bandwidth (*b*)	1.5 octaves			
aspect ratio (*γ*)	0.5			
(*c*) *cellular parameters*				
excit. cell somatic capacitance (*C*^E^_m_) and inhib. cell somatic capacitance (*C*^I^_m_)	500 pF, 214 pF			§
excit. cell somatic leakage conductance (*g*^E^_0_) and inhib. cell somatic leakage conductance (*g*^I^_0_)	25 nS, 18 nS			§
excit. cell membrane time constant (*τ*^E^_m_) and inhib. cell membrane time constant (*τ*^I^_m_)	20 ms, 12 ms			§
excit. cell resting potential (*V*^E^_0_) and inhib. cell resting potential (*V*^I^_0_)	−74 mV, −82 mV			§
excit. firing threshold potential (*Θ*^E^) and inhib. firing threshold potential (*Θ*^I^)	−53 mV, −53 mV			§
excit. after-spike hyperpolarization potential (*V*^E^_H_) and inhib. after-spike hyperpolarization potential (*V*^I^_H_)	−57 mV, −58 mV			§
absolute refractory period (*τ*_*R*_)	2 ms			§
(*d*) *synaptic parameters*				
synaptic neurotransmitter concentration (*α*_C_) and proportion of unblocked NMDA receptors (*α*_D_)	0.5			†
presynaptic STDP time constant (*τ*_C_) and postsynaptic STDP time constant (*τ*_D_)	{5, 25, 125} ms			†
synaptic learning rate (*ρ*)	0.1			†
range of synaptic conductance delay	[0.1, 10.0] ms			†
synaptic conductance scaling factor for FF excitatory connections from Gabor filters to layer 1 excit. cells (*λ*^GfE^ · Δ*g*^GfE^)	[0, 0.4] nS			*
synaptic conductance scaling factor for FF excit. connections to excit. cells in layers 2, 3 or 4 (*λ*^EfE^ · Δ*g*^EfE^)	[0, 1.6] nS			*
synaptic conductance scaling factor for FB excit. connections to excit. cells in layers 1, 2 or 3 (*λ*^EbE^ · Δ*g*^EbE^)	[0, 1.6] nS			*
synaptic conductance scaling factor for LAT excit. connections to excit. cells in layers 1, 2, 3 or 4 (*λ*^ElE^ · Δ*g*^ElE^)	[0, 1.6] nS			*
synaptic conductance scaling factor for LAT connections from excit. cells to inhib. cells in layers 1, 2, 3 or 4 (*λ*^ElI^ · Δ*g*^ElI^)	40 nS			*
synaptic conductance scaling factor for LAT connections from inhib. cells to excit. cells in layers 1, 2, 3 or 4 (*λ*^IlE^ · Δ*g*^IlE^)	80 nS			*
excitatory reversal potential ( 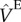 )	0 mV			§
inhibitory reversal potential ( 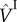 )	−70 mV			§
synaptic time constant for all FF, FB and LAT connections from Gabor filters and excit. cells to excit. cells (*τ*_GfE_, *τ*_EfE_, *τ*_EbE_, *τ*_ElE_)	150 ms			*
synaptic time constant for LAT connections from excit. cells to inhib. cells (*τ*_ElI_)	2 ms			§
synaptic time constant for LAT connections from inhib. cells to excit. cells (*τ*_IlE_)	5 ms			§
(*e*) *parameters for numerical simulation by forward Euler timestepping scheme*				
numerical step size (Δ*t*)	0.02 ms			

#### 3.1.2. Differential equations

The following subsections describe the cell, synapse and plasticity equations used in the simulations of [9], as well as the additional simulations described in this paper.

Cell equations

In the model developed by Eguchi *et al.* [9], each neuron is modelled as a conductance-based leaky integrate and fire (LIF) neuron. A neuron's membrane potential is updated according to3.1
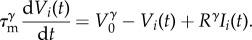


The cell membrane potential for a given neuron *V*_*i*_(*t*) (indexed by *i*) is driven up by current from excitatory conductance-based synapses, and down towards the inhibitory reversal potential by current from inhibitory conductance-based synapses. Neurons decay back to their resting state over a time course determined by the properties of their membrane. Here *τ*_m_ represents the membrane time constant, defined as *τ*_m_ = *C*_m_/*g*_0_, where *C*_m_ is the membrane capacitance, *g*_0_ is the membrane leakage conductance and *R* is the membrane resistance (*R* = 1/*g*_0_). *V*_0_ denotes the resting potential of the cell. Class-specific values (excitatory and inhibitory) are indexed by *γ* for the above neuron parameters. *I*_*i*_(*t*) represents the total current input from the afferent synapses (described in equation (3.2)).

The total synaptic current injected into a neuron is given by the sum of the conductances of all afferent synapses (excitatory and inhibitory), multiplied by the difference between the specific synapse class reversal potential (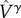) and the neuron membrane potential (*V*_*i*_(*t*)). The conductance of a given synapse is given by *g*_*ij*_, where *j* and *i* are the indices of the pre- and postsynaptic neurons, respectively,3.2
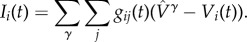


Synaptic conductance equations

The synaptic conductance of a particular synapse, *g*_*ij*_(*t*), is governed by a decay term *τ*_*g*_ and a Dirac delta function-based change (equation (3.4)) when spikes arrive from the presynaptic neuron *j* as follows:3.3
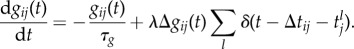


The conduction delay for a particular synapse is denoted by Δ*t*_*ij*_, which ranges from 0.1 to 10.0 ms, and each presynaptic neuron spike is indexed by *l*. A biological scaling constant *λ* has been introduced to scale the synaptic efficacy Δ*g*_*ij*_ which lies between unity and zero. The Dirac delta function is defined as follows:3.4
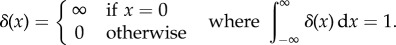


Synaptic learning equations

In the model investigated by Eguchi *et al.* [9], STDP operates at all of the bottom-up, top-down and lateral connections from excitatory cells to excitatory cells throughout layers 1–4. The equations for STDP at these excitatory–excitatory (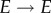) synapses were adapted from [29]. The form of STDP operating at a synaptic connection from presynaptic cell *j* to postsynaptic cell *i* is as follows.

The recent presynaptic activity, *C*_*ij*_(*t*), is modelled by3.5
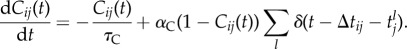
The variable *C*_*ij*_(*t*) represents the concentration of neurotransmitter (glutamate) released into the synaptic cleft [29] and is bounded by [0, 1] for 0 ≤ *α*_C_ ≤ 1. *C*_*ij*_(*t*) is governed by a decay term *τ*_C_ and is driven up by presynaptic spikes according to the model parameter *α*_C_. The inclusion of the axonal transmission delay Δ*t*_*ij*_ from presynaptic neuron *j* to postsynaptic neuron *i* in equation (3.5) ensures that *C*_*ij*_(*t*) is driven when the spike from presynaptic neuron *j* actually arrives at the postsynaptic neuron *i*.

The recent postsynaptic activity, *D*_*i*_(*t*), is governed by3.6
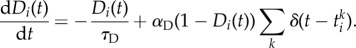
The variable *D*_*i*_(*t*) represents the proportion of *N*-methyl-D-aspartate (NMDA) receptors unblocked by recent depolarization from back-propagated action potentials [29]. *D*_*i*_(*t*) is governed by decay term *τ*_D_ and is driven up by postsynaptic spikes according to the model parameter *α*_D_. Postsynaptic neuron spikes are indexed by *k*.

The strength of the synaptic weight, Δ*g*_*ij*_(*t*), is governed by3.7
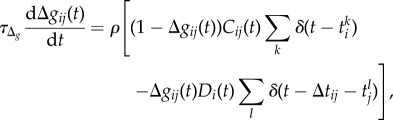
with time constant *τ*_Δ_*g*__.

The above STDP model operates as follows. If the variable representing presynaptic activity *C* is high (due to recent presynaptic spikes having arrived at the postsynaptic neuron) at the time of a postsynaptic spike, then the synaptic weight is increased (LTP). Alternatively, if the variable representing postsynaptic activity *D* is high (from recent postsynaptic spikes) at the time of a presynaptic spike arriving at the postsynaptic neuron, then the weight is decreased (LTD).

The model parameters used in the simulations performed by Eguchi *et al.* [9] were chosen to be as biologically accurate as possible and are given in table 1.

#### 3.1.3. Training the network on visual stimuli

In the simulations carried out by Eguchi *et al.* [9], visual images were first preprocessed by a set of Gabor filters that mimicked the responses of simple cells in V1 [32–34]. That is, individual Gabor filters responded to a bar or edge element with a particular orientation and retinal location. The outputs of the Gabor filters were used to set the firing rates of excitatory input neurons in layer 0. However, the actual spikes of the input cells were set to occur at randomized timings according to a Poisson distribution. So the original study did not impose any initial spatio-temporal structure on the spiking activity in layer 0.

The Gabor input filters used were computed by the following equation:3.8
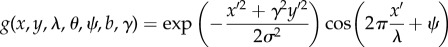
with the following definitions:3.9
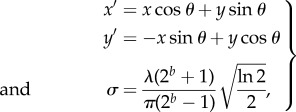
where *x* and *y* specify the position of a light impulse in the visual field [35]. The parameter *λ* is the wavelength (1/*λ* is the spatial frequency), *σ* controls the number of such periods inside the Gaussian window based on *λ* and spatial bandwidth *b*, *θ* defines the orientation of the feature, *ψ* defines the phase and *γ* sets the aspect ratio that determines the shape of the receptive field. In the experiments carried out by Eguchi *et al.* [9], an array of Gabor filters was generated at each of 128 × 128 retinal locations with the parameters given in table 1.

The outputs of the Gabor filters were used as the basis to generate Poisson spike trains as follows:3.10
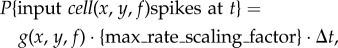
where *f* is the index of a Gabor filter used for the simulation and max_rate_scaling_factor is the maximum input neuron firing rate (set to 100 in the simulation studies). The outputs of the Gabor filters are used to set the firing rates of layer 0 excitatory input neurons. However, the spike times of the layer 0 neurons are randomized according to a Poisson distribution. The layer 0 neurons then propagate activity to the layer 1 excitatory neurons according to the synaptic connectivity given in table 1. That is, each layer 1 neuron receives connections from 30 randomly chosen layer 0 neurons localized within a topologically corresponding region of the retina. These connection distributions are defined by a radius shown in table 1.

### 3.2. Analysis of network performance

#### 3.2.1. Information analysis of average firing rate responses of single cells

Eguchi *et al.* [9] measured how much information is carried in the firing rates of cells in the fourth (output) layer of the network about the identity of visual stimuli presented to the model. If a neuron responds selectively with a high firing rate to only one particular stimulus, and responds to that stimulus across all transforms, then the firing rate response of the neuron carries maximal information about the presence of that visual stimulus. In the simulations performed in the original study, each presentation of a stimulus was considered a different transform because each stimulus presentation caused the input layer 0 neurons to emit a different randomized sequence of spikes according to the Poisson distribution. That is, the exact timings of the input neuron spikes were different for each presentation of the same stimulus. Hence different presentations of the same visual stimulus to the network were treated as ‘transforms’ of that stimulus.

The amount of stimulus-specific information that a specific cell carries is calculated using the following formula with details given by Rolls & Milward [36]:3.11
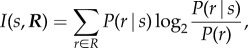
where *s* is a particular stimulus, *r* is the response of a cell to a single stimulus and ***R*** is the set of responses of a cell to the set of stimuli.

The maximum information that a cell could carry in its firing rate response is given by the formula3.12
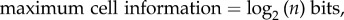
where *n* is the number of different visual stimuli.

#### 3.2.2. Information analysis of temporal spike patterns emitted by polychronous neuronal groups

Eguchi *et al.* [9] also applied information theory to quantify the amount of information carried by PNGs about the identity of visual stimuli presented to the network. However, to simplify the analysis, the authors only investigated the information carried by simple PNGs consisting of two spikes emitted by a pair of neurons.

The original study used the spike trains recorded during multiple stimulus presentations to the network to compute the probabilities that a given spike-pair will occur in response to the presentation of each of the stimuli *s*. These probabilities are based on the frequency of occurrence of the spike-pair across multiple transforms (presentations) of each stimulus *s*. From these frequency distributions the following probability table for each stimulus category *s* was constructed:3.13
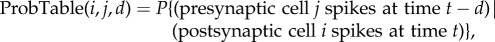
where *i* and *j* are the indices of two neurons under consideration, *t* is the time at which the cell *i* emits a spike and *d* is the time interval that neuron *i* emits a spike after neuron *j*. Eguchi *et al.* [9] considered values of *d* within the range of [0, 10 ms], where this time interval was divided into 10 equal bins of 1 ms.

The original study then applied the information analysis methodology of §3.2.1 to analysing the information carried by spike-pair PNGs, and in doing so regarded the probability table given by equation (3.13) as ***R***, the set of responses to the set of stimuli, used in equation (3.11). Thus, equation (3.11) was used to compute the information carried by spike-pair PNGs about the presence of a particular stimulus *s*. Using this approach, the authors were able to quantify how selective such spike-pair PNGs were for particular stimuli. If a particular spike-pair PNG responds invariantly to the transforms (presentations) of a particular stimulus *s* but not to the other stimuli, then the spike-pair PNG carries maximum information about the presence of its preferred stimulus.

## 4. Performance of spiking neural network models

### 4.1. The emergence of polychronization through successive network layers

We begin by presenting some new simulation results from a simplified two-layer bottom-up (feedforward) spiking neural network model. These simulation results take a more detailed look at the gradual emergence of polychronization through successive layers than was carried out by Eguchi *et al.* [9]. The results presented can be contrasted with simulations carried out Diesmann *et al.* [19], which demonstrated the emergence of synchronization through successive layers of spiking neurons. For synchronization to emerge, it was necessary for Diesmann *et al.* [19] to incorporate either no axonal transmission delays or axonal delays all of the same length. In the new simulations presented here we show that incorporating randomized distributions of axonal delays, say spread in the interval [1, 30] ms, into the bottom-up connections flips the model from synchronous to polychronous behaviour. This important mechanism, in turn, permits the emergence of binding neurons embedded within these polychronous stimulus representations, as described elsewhere in this paper.

**Figure 7.**
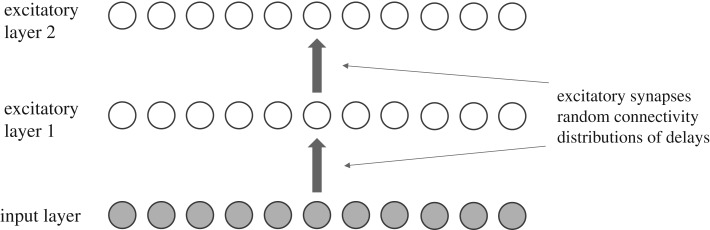
A two-layer feedforward spiking neural network model. The model consists of a one-dimensional input layer consisting of 1000 excitatory neurons. The spike times of active input layer neurons are taken from a Poisson distribution (equation (3.10)) with a mean firing rate of 50 Hz. The input layer sends bottom-up synaptic connections to layer 1, which in turn sends connections to the output layer 2. Layers 1 and 2 each consist of 1000 LIF excitatory spiking neurons. The bottom-up connections to layers 1 and 2 are modified during learning according to the STDP rule implemented by Eguchi *et al.* [9]. Neurons in the first LIF layer receive connections from the input layer neurons with a connection probability of 0.2, while neurons in the second LIF layer receive connections from neurons in the first LIF layer with a probability of 0.02. Axonal transmission delays between the input layer and the first LIF layer are uniformly distributed between 1 and 10 ms, while axonal delays between the first LIF layer and the second LIF layer are uniformly distributed between 1 and 30 ms. Copyright © 2018 American Psychological Association. Reproduced [or Adapted] with permission. The official citation that should be used in referencing this material is [9]. No further reproduction or distribution is permitted without written permission from the American Psychological Association.

The two-layer neural network model simulated is shown in figure 7. The model consists of a one-dimensional input layer consisting of 1000 excitatory neurons. The spike times of active input layer neurons are taken from a Poisson distribution (equation (3.10)) with a mean firing rate of 50 Hz. The input layer sends bottom-up synaptic connections to layer 1, which in turn sends connections to the output layer 2. Layers 1 and 2 each consist of 1000 LIF excitatory spiking neurons. The bottom-up connections to layers 1 and 2 are modified during learning according to the STDP rule implemented by Eguchi *et al.* [9]. The equations governing the cellular and synaptic dynamics, including synaptic plasticity, are given in §3.1.2. Neurons in the first LIF layer receive connections from the input layer neurons with a connection probability of 0.2, while neurons in the second LIF layer receive connections from neurons in the first LIF layer with a probability of 0.02. Axonal transmission delays between the input layer and the first LIF layer are uniformly distributed between 1 and 10 ms, while axonal delays between the first LIF layer and the second LIF layer are uniformly distributed between 1 and 30 ms. All neuron, synapse and learning parameters that are not described in this subsection are the same as the values originally used by Eguchi *et al.* [9] shown in table 1. We refer to this model as a two-layer model because there are two layers of LIF spiking neurons that receive plastic bottom-up connections, which are modified during training.

The network is trained and tested with a single input stimulus, which is represented by activating all of the neurons in the input layer. Each of the activated input neurons has its average spike rate set to 50 Hz.

The response of the network to the stimulus is initially tested before training. Ten such simulations are run, in each of which the stimulus is applied to the input layer and activity allowed to propagate up through layers 1 and 2. Each such simulation activates the same stimulus pattern in the input layer, but uses a different seed to generate the spikes according to a Poisson distribution. This ensures that the spike times of the activated input neurons are randomized across the simulations. We identified which neurons in the higher layers 1 and 2 responded to the stimulus across all 10 simulations. For each such neuron, the time of its first spike after the stimulus was presented was recorded in each of the simulations. Then, for each neuron, we calculated the mean and standard deviation of the time of its first spike over the 10 stimulus presentations. The standard deviation of the first spike times provided a measure of the amount of temporal variation in the neuron's spike response.

The network is then trained by presenting the single stimulus to the network 10 times using different seeds to generate the Poisson input spike times on each presentation. During training, synaptic weights are updated using the equations described in §3.1. The STDP time constants are set to *τ*_C_ = 100 ms and *τ*_D_ = 150 ms, while the learning rate is set to *ρ* = 0.1.

After training, the network is again tested by running 10 separate simulations as described above for the pretraining test.

**Figure 8.**
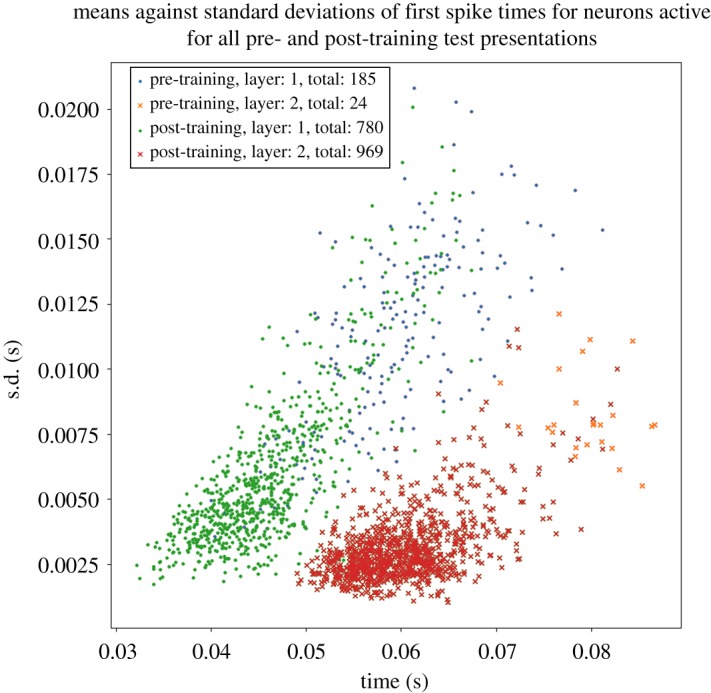
Scatterplot showing the emergence of polychronous spatio-temporal structure in neuronal spike times after training and through successive network layers. Each marker corresponds to an individual neuron in either of the higher layers 1 or 2 that was activated by the stimulus across all test presentations. The following four sets of simulation results are presented: pretraining layer 1 neurons (blue dots), pretraining layer 2 neurons (orange crosses), post-training layer 1 neurons (green dots) and post-training layer 2 neurons (red crosses). For each neuron, the mean time of its first spike across all 10 simulations in which a stimulus is presented (abscissa) is plotted against the standard deviation in these first spike times (ordinate). In these simulations, the axonal transmission delays between the input layer and layer 1 are uniformly distributed between 1 and 10 ms, while axonal delays between layer 1 and layer 2 are uniformly distributed between 1 and 30 ms. It is evident that training the network leads to a significant reduction in the standard deviations of first spike times in layers 1 and 2. Thus, training the network using STDP reduces the degree of temporal variation in the first spike times. Moreover, layer 2 neurons have reduced standard deviations in their first spike times compared with layer 1 both before and after training. So successive layers of processing also reduce the degree of temporal variation in the first spike times as hypothesized. Copyright © 2018 American Psychological Association. Reproduced [or Adapted] with permission. The official citation that should be used in referencing this material is [9]. No further reproduction or distribution is permitted without written permission from the American Psychological Association.

Figure 8 shows the emergence of polychronization after training and through successive network layers. Each marker in the scatter plot corresponds to an individual neuron in the higher layers 1 and 2 that was activated by the stimulus across all test presentations. Four sets of simulation results are presented as follows: pretraining layer 1 neurons (blue dots), pretraining layer 2 neurons (orange crosses), post-training layer 1 neurons (green dots) and post-training layer 2 neurons (red crosses). For each neuron, the mean time of its first spike across all 10 simulations in which a stimulus is presented (abscissa) is plotted against the standard deviation in these first spike times (ordinate). In these simulations, the axonal transmission delays between the input layer and layer 1 are uniformly distributed between 1 and 10 ms, while axonal delays between layer 1 and layer 2 are uniformly distributed between 1 and 30 ms. First, it can be seen that the number of neurons spiking on all test presentations increases after training for both layers 1 and 2. It is also evident that training the network leads to a significant reduction in the standard deviations of first spike times in layers 1 and 2, demonstrating an increase in temporal precision after training. Moreover, the standard deviations are lower in the second layer than in the first layer, demonstrating that the emergence of polychronization takes place inductively over layers as hypothesized. Interestingly, the mean first spike times of the second layer neurons are not synchronous but are instead spread out in time (polychronous). The incorporation of broad distributions of axonal delays into the network ensures that individual neurons in the higher layers emit their first spikes at different times with respect to each other, thus giving rise to the emergence of polychronization.

**Figure 9.**
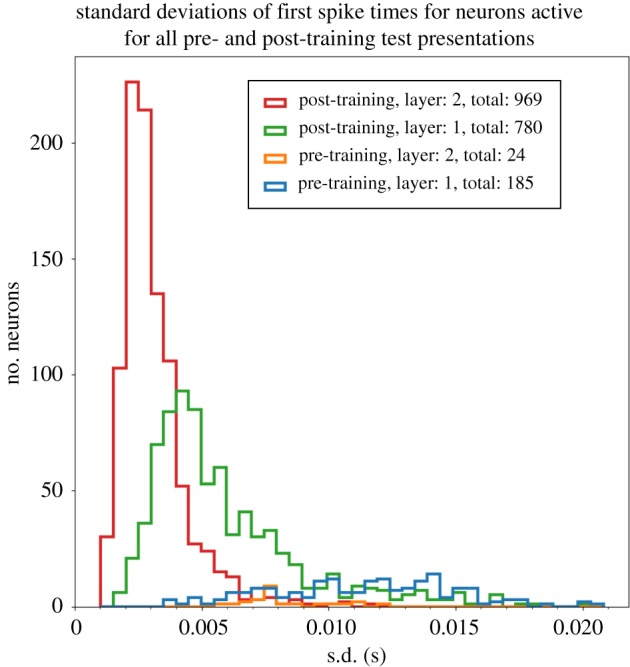
Histogram showing the frequency distributions of standard deviations in the first spike times of neurons in layers 1 and 2 in response to a stimulus presentation. This figure represents the same simulation data as in figure 8. For each neuron, we compute the standard deviation in its first spike across all 10 simulations in which a stimulus is presented. The following four sets of simulation results are presented: pretraining layer 1 (blue line), pretraining layer 2 (orange line), post-training layer 1 (green line) and post-training layer 2 (red line). In these simulations, the axonal transmission delays between the input layer and layer 1 are uniformly distributed between 1 and 10 ms, while axonal delays between layer 1 and layer 2 are uniformly distributed between 1 and 30 ms. As in figure 8, it can be seen that training the network using STDP reduces the degree of temporal variation in the first spike times, and that successive layers of processing also reduce the degree of temporal variation.

Figure 9 shows the frequency distributions of standard deviations in the first spike times of neurons in layers 1 and 2 in response to a stimulus presentation. This figure represents the same simulation data as shown in figure 8. As in figure 8, it can be seen that the temporal precision in the first spike times is increased by both training the network and through successive layers of processing.

**Figure 10.**
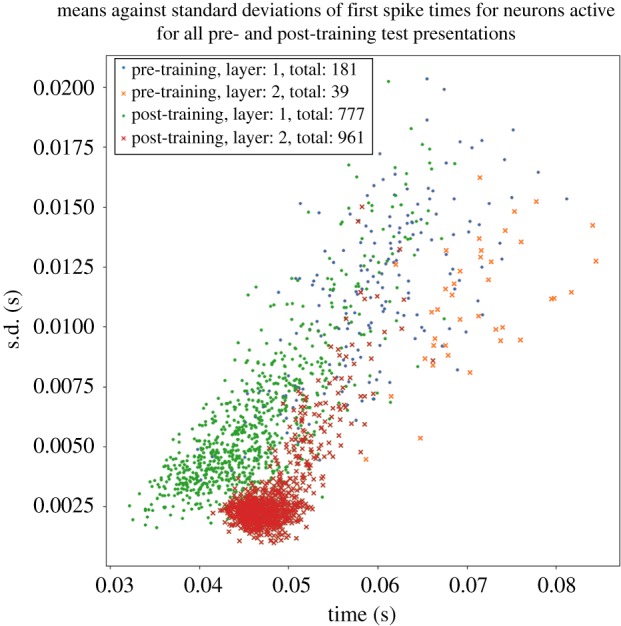
Performance of network with 1 ms axonal transmission delays between layer 1 and layer 2. Plot styled as in figure 8. It is evident that with uniform axonal delays of 1 ms in all connections from layer 1 to layer 2 the mean spike times in layer 2 are much more synchronous (clustered close together in time) than the behaviour (spread out in time) seen in figure 8 with a broad distribution of axonal delays in the interval [0, 30] ms. Hence, comparing these results shows that incorporating a broad distribution of axonal delays drives the system to reliable spiking over a greater temporal range.

Figure 10 shows the performance of the network with uniform axonal delays of 1ms in all connections from layer 1 to layer 2. The plot is styled as in figure 8. It is evident that the mean spike times in layer 2 are much more synchronous (clustered close together in time) than the polychronous behaviour (spread out in time) seen in figure 8. Hence, comparing these results shows that increasing the range of axonal transmission delays between layer 1 and layer 2 makes the spike times of the layer 2 neurons polychronous rather than synchronous.

### 4.2. Selected simulation results from Eguchi *et al.* [9]

In this section, we review some of the simulation results from Eguchi *et al.* [9]. The simulation study carried out by these authors demonstrated the emergence of stimulus-specific PNGs, binding neurons, and the bottom-up projection of visual information about lower-level features to the highest network layers.

#### 4.2.1. Training and testing the network model on a set of visual stimuli

**Figure 11.**
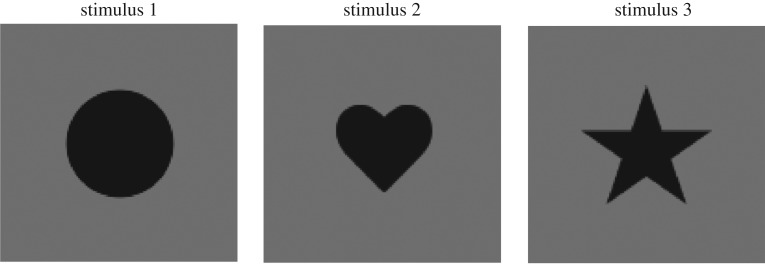
The set of three visual stimuli presented to the network during training and testing in the simulation study of Eguchi *et al.* [9]. The stimulus set included a circle, a heart and a star. Reproduced with permission from Eguchi *et al.* [9].

Eguchi *et al.* [9] trained and tested their network on the three visual stimuli shown in figure 11, which included a circle, a heart and a star.

During the initial training phase, the three stimuli were repeatedly presented in turn to the network. At each stimulus presentation, the Gabor filters (equation (3.8)), which mimic the responses of bar/edge-detecting simple cells in cortical visual area V1, were convolved with the image of the stimulus. The outputs of the Gabor filters were used to set the firing rates of the input neurons in layer 0. Crucially, the spike times of the input neurons were randomized according to a Poisson distribution (equation (3.10)). These spikes were then propagated up through the network layers according to the model equations described in §3.1. As the visual signals propagated through the network, the plastic excitatory connections (which included the connections from the Gabor filters to layer 1 excitatory neurons, as well as the bottom-up, top-down and lateral connections between excitatory neurons across layers 1–4) were modified according to the STDP learning rule (3.7).

When testing the model, the same three stimuli were presented to the network with STDP turned off. For each stimulus presentation, the spike train responses of all fourth (output) layer neurons were recorded.

#### 4.2.2. Stimulus information carried by the average firing rates of neurons and spike-pair PNGs in the output layer of the network

Eguchi *et al.* [9] analysed the stimulus information carried either in the average firing rates of fourth (output) layer neurons or the spike-pair PNGs in the output layer. The maximum amount of information that can be carried by a single neuron or spike-pair PNG is log_2_(*n*), where *n* is the number of stimuli. In these simulations, there were three stimuli as shown in figure 11. Therefore, the maximum possible information is log_2_(3) ≈ 1.58 bits. Network performance was investigated with different combinations of feedforward (FF), feed-back (FB) and lateral (LAT) connectivity between excitatory neurons in layers 1–4. Specifically, the original study presented results for the full network architecture FF + FB + LAT before training, and results after training with the following different forms of connectivity: FF, FF + FB, FF + LAT, FF + FB + LAT.

**Figure 12.**
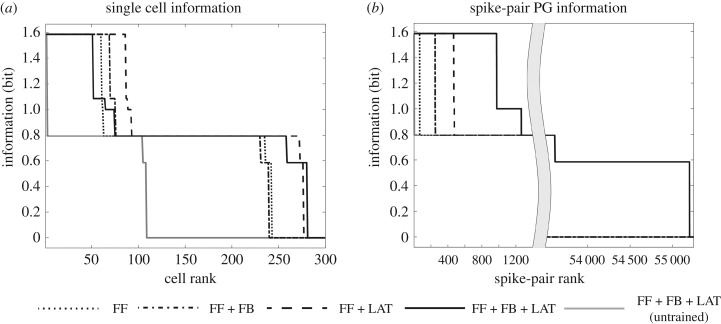
(*a*) Stimulus information carried in the average firing rates of fourth (output) layer neurons. Eguchi *et al.* [9] analysed the information carried in the average firing rates of fourth (output) layer neurons according to the procedure described in §3.2.1. They investigated network performance with different combinations of feedforward (FF), feed-back (FB) and lateral (LAT) connectivity between excitatory neurons in layers 1–4. Results after training are shown for network architectures with the following kinds of synaptic connectivity: FF, FF+FB, FF+LAT, FF+FB+LAT. Results before training are shown only for the full network architecture FF+FB+LAT. For each plot, the maximum single cell information carried by 300 output neurons is shown, where the neurons are arranged along the abscissa in rank order. It is evident that very few output neurons in the FF+FB+LAT model reached the maximal information of 1.58 bits before training. However, after training, all four network architectures developed about 50–100 neurons with maximal stimulus information. (*b*) Stimulus information carried by spike-pair PNGs in the output layer. Eguchi *et al.* [9] also analysed the information carried by spike-pair PNGs in the output layer according to the procedure described in §3.2.2. It can be seen that the full network architecture with FF+FB+LAT connections produced the most spike-pair PNGs with maximal information. The full network architecture has developed almost 1000 spike-pair PNGs that carry the maximum information of 1.58 bits. These simulation results thus demonstrate the large-scale emergence of stimulus-specific PNGs after training the network on the set of visual stimuli shown in figure 11. Reproduced with permission from Eguchi *et al.* [9]. Copyright © 2018 American Psychological Association. Reproduced [or Adapted] with permission. The official citation that should be used in referencing this material is [9]. No further reproduction or distribution is permitted without written permission from the American Psychological Association.

Figure 12*a* shows the stimulus information carried in the average firing rates of fourth (output) layer neurons. For each plot, the single cell information carried by 300 output neurons is shown, where the neurons are arranged along the abscissa in rank order. It is evident that very few output neurons in the FF + FB + LAT model reached the maximal information of 1.58 bits before training. However, after training, all four network architectures developed 50–100 neurons with maximal stimulus information. Nevertheless, it is evident that the network incorporating all three categories of connections, which is closer to the connectivity observed in the visual cortex, gave the lowest performance when analysing the information carried by the average firing rates of neurons.

Figure 12*b* shows the information carried by spike-pair PNGs in the output layer. It can be seen that the full network architecture with FF + FB + LAT connections produced the most spike-pair PNGs with maximal information. The full network architecture has developed almost 1000 spike-pair PNGs that carry the maximum information of 1.58 bits. These simulation results thus demonstrate the large-scale emergence of stimulus-specific PNGs after training the network on the set of visual stimuli shown in figure 11. In contrast to the results shown in figure 12*a*, when analysing the stimulus information carried by the spike-pair PNGs, the best performance is achieved by the full network architecture incorporating FF + FB + LAT connections, which is closest to the actual architecture of the visual cortex.

Interestingly, the number of spike-pair PNGs with maximal stimulus information that emerged after training in the full network architecture was about an order of magnitude greater than the number of output neurons that carried maximal stimulus information in their average firing rates. This occurred even though the stimulus representations in the input layer 0 had randomized spike times set according to a Poisson distribution. In this case, the polychronous neuronal activity emerged naturally and robustly through the hierarchy of network layers. Eguchi *et al.* [9] concluded that these observations provided evidence for the potential presence of polychronous stimulus representations in the visual brain, which may also offer increased representational capacity.

However, as discussed in §1, there is a potential issue with the simulation results of the original study showing the emergence of spike-pair PNGs carrying high levels of stimulus-specific information. Specifically, if two neurons respond selectively to a preferred stimulus with high firing rates and do not respond to any other stimuli, then it may be possible to identify spike-pair PNGs that appear to carry high levels of stimulus-specific information even if the spike times are random. Because of this, we have presented new simulation results taking a closer look at the emergence of polychronization through a hierarchy of network layers in §4.1.

#### 4.2.3. How the stimulus information carried by spike-pair PNGs in the output layer is affected by key model parameters

Eguchi *et al.* [9] explored how the stimulus information carried by spike-pair PNGs in the output layer is affected by varying two important model parameters: the STDP time constants and the number of synaptic contacts between each pair of pre- and postsynaptic neurons. This part of their investigation used the full network architecture with all three kinds of synaptic connectivity, that is, FF + FB + LAT.

**Figure 13.**
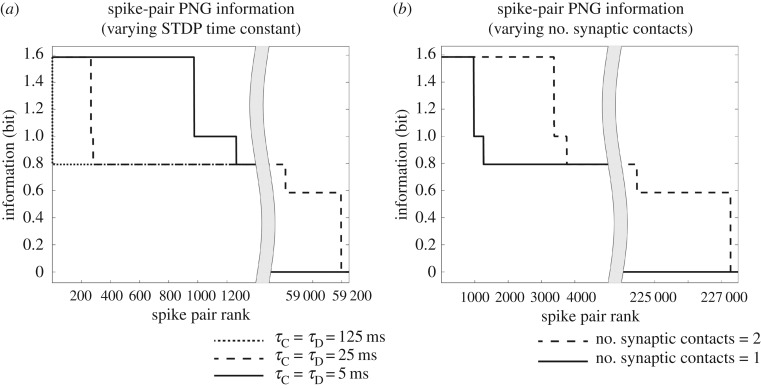
(*a*) Effect of varying the STDP time constants. Eguchi *et al.* [9] analysed how the stimulus information carried by spike-pair PNGs in the output layer was affected by varying the STDP time constants across the range *τ*_C_ = *τ*_D_ = 125 ms, 25 ms or 5 ms. It was found that the network developed the most spike-pair PNGs carrying maximal information about stimulus identity when the STDP time constants were shortest. Short STDP time constants maintain the temporal precision of the STDP, which is required to promote the development of stimulus-specific PNGs. (*b*) Effect of varying the number of synaptic connections between each pair of pre- and postsynaptic excitatory neurons. The original study also analysed how varying the number of synaptic connections between each pair of pre- and postsynaptic neurons affected the stimulus information carried by spike-pair PNGs in the output layer. Results are shown for simulations with either one or two connections between each pair of pre- and postsynaptic neurons. In the case of two synaptic connections, the contacts are assigned different axonal delays chosen randomly in the interval [0, 10] ms. This allows the STDP to selectively strengthen one connection with a particular axonal delay instead of the other connection with a different delay in order to promote the development of PNGs within the network. Indeed, it can be seen that far more PNGs carrying maximal stimulus information emerge in the simulations with two connections between each pair of pre- and postsynaptic neurons. Copyright © 2018 American Psychological Association. Reproduced [or Adapted] with permission. The official citation that should be used in referencing this material is [9]. No further reproduction or distribution is permitted without written permission from the American Psychological Association.

Figure 13*a* shows how the stimulus information carried by spike-pair PNGs in the output layer was affected by varying the STDP time constants. In the results shown, the STDP time constants were varied over the values *τ*_C_ = *τ*_D_ = 125 ms, 25 ms or 5 ms. It was found that many more spike-pair PNGs carrying maximal information about stimulus identity emerged in the network when the STDP time constants were shortest. Short-duration STDP time constants are needed to maintain the temporal precision of the STDP, which is in turn required to promote the development of stimulus-specific PNGs. However, as the STDP time constants are increased, the synaptic plasticity becomes less dependent on the precise timings of spikes, and begins to operate more like a classical rate-coded Hebbian learning rule. In this case, the emergence of stimulus-specific PNGs is degraded.

Figure 13*b* shows how varying the number of synaptic connections between each pair of pre- and postsynaptic excitatory neurons affected the stimulus information carried by spike-pair PNGs in the output layer. The underlying hypothesis here is that a presynaptic neuron might make multiple synaptic contacts on a postsynaptic neuron, perhaps as the axon from the presynaptic neurons weaves its way through the dendritic tree of the postsynaptic neuron, and that each of these synaptic contacts might have a somewhat different axonal/synaptic transmission delay associated with it. Could STDP then selectively strengthen one (or a small subset) of these connections with a particular transmission delay instead of the other connections with different transmission delays in order to promote the development of polychronous stimulus representations? Results are shown in figure 13*b* for simulations with either one or two connections between each pair of pre- and postsynaptic neurons. It is evident that far more PNGs carrying maximal stimulus information emerge in the simulations with two connections between each pair of pre- and postsynaptic neurons. Thus, as hypothesized, having multiple (e.g. two) synaptic contacts with different transmission delays between each pair of pre- and postsynaptic excitatory neurons permits STDP to selectively strengthen the connection (or subset of connections) with a particular transmission delay that will enhance the emergence of stimulus-specific PNGs.

**Figure 14.**
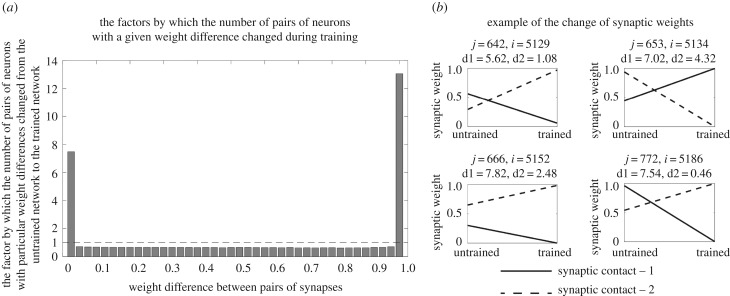
Examples of how STDP selectively strengthens connections with particular delays between pairs of pre- and postsynaptic neurons with multiple synaptic contacts. Eguchi *et al.* [9] showed results for a model in which each pair of pre- and postsynaptic neurons had two connections with different randomly assigned transmission delays in the interval [0, 10] ms. The figure shows how the strengths of the two synaptic connections with different delays between four example pairs of pre- and postsynaptic neurons are modified by STDP during visual training. It can be seen that, in each of the four cases, one connection is selectively strengthened, while the other is weakened. In this way, STDP is able to effectively choose which transmission delay to strengthen in the connectivity between the two neurons in order to promote the emergence of polychronization within the network. Reproduced with permission from Eguchi *et al.* [9].

Figure 14 shows how STDP selectively strengthens connections with particular delays between pairs of pre- and postsynaptic neurons with multiple synaptic contacts. Results are presented for simulations in which each pair of pre- and postsynaptic excitatory neurons has two synaptic contacts with different transmission delays randomly chosen from the interval [0, 10] ms. For each pair of neurons, the corresponding plot shows that one synaptic connection with a particular delay is strengthened, while the other connection with a different delay is weakened. Thus, it can be seen that STDP selectively strengthens or weakens synaptic connections according to the durations of their transmission delays during visual training. This, in turn, helps to promote the emergence of stimulus-specific PNGs.

#### 4.2.4. The development of binding neurons during visual training

**Figure 15.**
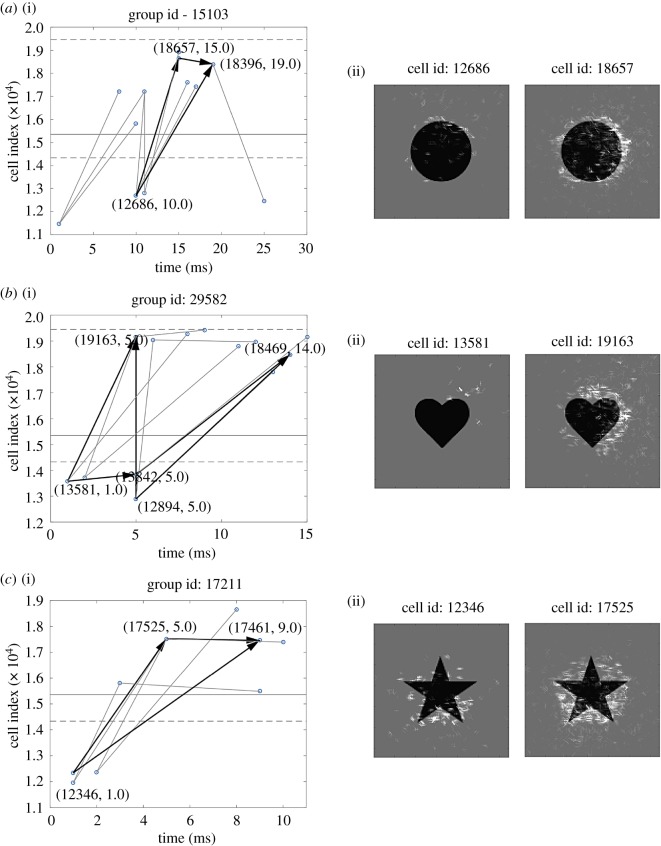
Examples of binding neurons that develop during visual training. Eguchi *et al.* [9] showed the emergence of three-neuron binding circuits in the full network architecture, including FF+FB+LAT connections, after training on the circle, heart and star shown in figure 11. Each row shows an example of a stimulus-selective PNG. (*a*(i),*b*(i),*c*(i)) The neurons in the PNG, where the neurons are represented by circles and the strengthened connections between the neurons are represented by lines. The neurons are plotted along the abscissa according to the relative timings of their spikes within the PNGs, which was determined by the axonal transmission delays of the strengthened connections between the neurons. (*a*(ii),*b*(ii),*c*(ii)) The patterns of input Gabor filters with the strongest bottom-up connectivity to the lower- and higher-level feature neurons shown in (*a*(i),*b*(i),*c*(i)). It can be seen that rows (*a*) and (*c*) represent examples of the hypothesized three-neuron binding circuits illustrated in figures 3*a* and 4*a*. Reproduced with permission from Eguchi *et al.* [9].

Eguchi *et al.* [9] demonstrated that embedded within the stimulus-specific PNGs that emerged in the full network architecture during training on the circle, heart and star shown in figure 11 were binding neurons of the kind illustrated in figures 3*a* and 4*a*. Simulation results are presented in figure 15. Each row shows an example of a stimulus-selective PNG, where the PNGs shown in rows (*a*)–(*c*) respond selectively to the circle, heart and star, respectively. Each row shows an example of a stimulus-selective PNG. Figure 15*a*(i),*b*(i),*c*(i) shows the neurons in the PNG, where the neurons are represented by circles and the strengthened connections between the neurons are represented by lines. The neurons are plotted along the abscissa according to the relative timings of their spikes within the PNGs, which was determined by the axonal transmission delays of the strengthened connections between the neurons. The right plots show the patterns of input Gabor filters with the strongest bottom-up connectivity to the lower- and higher-level feature neurons shown in figure 15*a*(i),*b*(i),*c*(i).

Rows (*a*) and (*c*) of figure 15 represent examples of the hypothesized three-neuron binding circuits illustrated in figures 3*a* and 4*a*. For example, consider the three-neuron binding circuit illustrated in row 3. Neuron 12686 is situated in layer 3 and represents the lower-level feature, neuron 18657 is situated in the output layer 4 and represents the higher-level feature, and neuron 18396 is a binding neuron that represents the binding relationship between the lower- and higher-level features. It can be seen that the axonal transmission delay from the lower-level feature neuron 12686 to binding neuron 18396 is equal to the transmission delay from the lower-level feature neuron 12686 to higher-level feature neuron 18657 plus the transmission delay from the higher-level feature neuron 18657 to binding neuron 18396. Given this pattern of axonal transmission delays between the three neurons, the spikes emitted by the lower-level feature neuron 12686 and higher-level feature neuron 18657 will arrive simultaneously at, and hence fire, binding neuron 18396 if and only if the lower-level feature neuron 12686 is actually participating in firing the higher-level feature neuron 18657. Row (*c*) presents another similar example of a three-neuron binding circuit. Figure 15*a*(ii),*c*(ii) confirm that the layer 3 neurons (left) represent lower-level features, while the layer 4 neurons (right) represent higher-level features of their preferred visual stimuli.

#### 4.2.5. Bottom-up projection of visual information about lower-level features to higher layers of the network

The simulations of Eguchi *et al.* [9] demonstrated the bottom-up propagation of visual information about lower-level features to the higher network layers according to the holographic principle described in §2.3 and illustrated in figure 4*a*. In the examples of three-neuron binding circuits shown in figure 15*a*,*c*, the lower-level feature neuron is situation in layer 3, the higher-level feature neuron is situated in layer 4 and the binding neuron is located in layer 4. Thus, the binding neuron is located in the same layer as the higher level feature neuron. The simulation results shown in figure 15 are examples of the kind of bottom-up projection of visual information shown in figure 4*a*. This kind of bottom-up projection of visual information about lower-level features to the higher network layers could make more fine-grained visuospatial information available at the end of the visual pathway for readout by subsequent brain areas involved in decision-making and behaviour.

## 5. Discussion

In this paper, we have discussed a new approach to solving the feature-binding problem in visual neuroscience that relies on the emergence of polychronization within biological spiking neural networks. This problem is described by authors in different ways, but broadly refers to the ability of the visual brain to represent the hierarchical relationships between lower- and higher-level visual features within a scene. Solving this problem is essential for understanding how the brain builds an integrated and coherent representation of the visual world. We suggest that solving how feature binding is accomplished by the brain will be necessary for the future development of artificial general intelligence and machine consciousness.

Simulation studies carried out by Eguchi *et al.* [9] have reported that fixed spatio-temporal patterns of spikes emerge automatically within the higher layers of a spiking neural network, and are repeated across different presentations of the same stimulus, even when the stimulus input representations have entirely randomized spike timings. These authors investigated the emergence of both large-scale PNGs consisting of many neurons and spike-pair PNGs consisting of just two neurons that carried high levels of stimulus-specific information. However, as discussed in §1, there is a potential issue with the latter results. That is, if two neurons respond with high firing rates to a preferred stimulus, but do not respond to any other stimuli, then it would still be possible to find spike-pair PNGs that carry high levels of stimulus-specific information in a random spike train. Consequently, given this possibility, in §4.1 we have presented some new simulation results that take a closer look at the emergence of polychronization through successive network layers. These simulation results show how precise spatio-temporal spike patterns may emerge naturally and automatically in the higher layers even though the input stimulus patterns have randomized spike times.

The hypothesis that such PNGs might develop was strongly inspired by the work of Diesmann *et al.* [19], which showed the emergence of synchronization in the higher layers of a hierarchical feedforward spiking neural network. For Diesmann *et al.* [19] to demonstrate the emergence of synchrony, these authors had to implement either no axonal transmission delays or axonal delays of the same duration. However, in the brain axonal transmission delays within the visual cortex are not all of the same duration. The key result introduced by Eguchi *et al.* [9] and further investigated in this paper is that incorporating distributions of axonal delays, say in the interval [0, 10] ms, flips the network behaviour from the emergence of synchronization in the higher layers to the emergence of polychronization. Consistent with this theoretical result, neurophysiology studies have observed the presence of polychronous activity in the brain [13,23,24].

**Figure 16.**
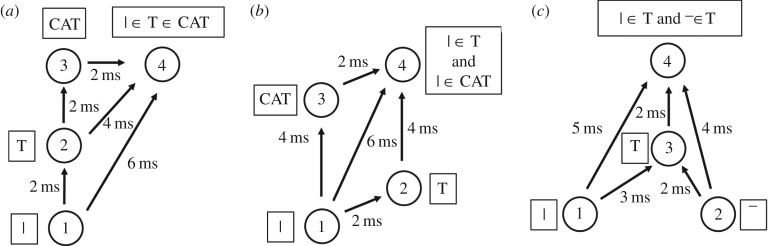
More complex kinds of feature-binding representation. This figure shows three examples of binding circuits that encode more complex forms of hierarchical binding relationship between features. (*a*) The binding neuron responds when a lower-level feature such as a vertical bar is part of an intermediate-level feature such as the letter T, which is in turn part of a higher-level feature such as the word CAT. (*b*) The binding neuron responds when a low-level feature such as a vertical bar is simultaneously part of two different higher-level features such as the letter T and the word CAT. (*c*) The binding neuron responds when two lower-level features such as a vertical bar and a horizontal bar are both part of a higher-level feature such as the letter T. Reproduced with permission from Eguchi *et al.* [9].

Why is polychronization important? When Eguchi *et al.* [9] trained their spiking neural network model of the primate ventral visual pathway using STDP to modify the synaptic connections, they reported seeing the emergence of polychronous stimulus representations. In particular, embedded within these PNGs were feature-binding neurons that represented the hierarchical binding relationships between lower- and higher-level visual features. These authors reported the emergence of three-neuron binding circuits in the general form illustrated in figure 3*a*. These kinds of feature-binding representations could emerge simultaneously at every level of the hierarchy of network layers, which encode visual features at different spatial scales, and everywhere across the visual field. However, the three-neuron binding circuits shown in figure 3*a* are only the simplest possible realization of the basic approach to feature binding using polychronization. For example, many other kinds of more complex feature-binding representations may emerge such as those illustrated in figure 16. Moreover, as illustrated in figure 3*b*, the representations of the lower- and higher-level features, as well as the feature-binding representations, may also take the form of PNGs. Furthermore, the connectivity between these features and feature-binding representations could be poly-synaptic instead of the simple mono-synaptic connectivity shown in figure 3*a*. It is quite clear, then, that we are at the beginning of exploring the nature of the polychronous representations of features and feature-binding relationships that may emerge within spiking neural networks.

Eguchi *et al.* [9] also proposed that information about lower-level visual features could be projected upwards to the higher network layers, where it would be available for subsequent brain systems involved in decision-making and behaviour. The simplest way in which this was hypothesized to occur was illustrated in figure 4*a*. This proposed mechanism was demonstrated in the simulation results shown in figure 15*a*,*c*. Here it could be seen that the binding neuron representing the hierarchical relationship between a lower-level feature and a higher-level feature emerged in the same higher layer as the neuron representing the higher-level feature. In this way, information about the lower-level feature, including its binding relationship to the higher-level feature, was projected up to the same higher layer representing the higher-level feature. However, many other more complex circuit architectures could develop during visual training that could project visual information about lower-level features upward to higher network layers as shown in figure 4*b*. Experimental evidence for the upward projection of fine-grained visuospatial information to higher brain areas has been provided by neurophysiology studies in monkeys. The PFC is a brain area that is strongly implicated in decision-making and behaviour. It receives inputs from the end of the ventral visual pathway. Rainer *et al.* [18] showed that information about the location of a target object was encoded in the responses of neurons in the PFC. This observation implies that visual neurons in the PFC encode the spatial configuration of objects rather than just the identity of the whole objects themselves.

An outstanding question is how later decision-making areas of the brain, such as the PFC, might readout and use visual information encoded by PNGs in the visual cortex. Given fast synaptic time constants, the responses of real neurons in the brain will be sensitive to the timings of incoming spikes. In particular, a postsynaptic neuron will be more likely to fire if the afferent spikes from a subpopulation of presynaptic neurons arrive at the postsynaptic neuron near simultaneously. Given the presence of random axonal transmission delays between neurons, say within the interval [0, 10] ms, the postsynaptic neuron will have the greatest probability of firing when the presynaptic neurons emit their spikes in a specific spatio-temporal sequence that ensures the spikes arrive at the postsynaptic neuron together. Thus, PNGs would appear to be the natural way in which neurons should be expected to encode information in the visual brain for subsequent readout by decision-making brain areas. Furthermore, the holographic principle proposes that information about visual features at all spatial scales, including the binding relations between these features, is projected upwards to such decision-making areas. Evidence for this emerged in the neural network simulations of Eguchi *et al.* [9]. Future experimental studies may investigate whether such an upward projection of visual information occurs in the brain by analysing the visuospatial information present in the PFC about not only the identity of visual objects but also the detailed spatial structure of these objects. For example, single/multi-unit recording studies in the PFC area of the monkey brain could test for the presence of visual neurons that encode the parts of objects as well as their spatial relationships with the whole object. The upward projection of such detailed visuospatial information to brain systems that produce behavioural responses is consistent with the hierarchical phenomenology of human vision described by Duncan & Humphreys [4]. Obviously, such hierarchical visual representations are useful, and in fact essential, for guiding behaviour in natural spatial environments. Moreover, it has been known since the early experimental studies of Edward Tolman [37] that even non-primates such as rats naturally learn about the structure of their environment and produce behaviour that seems to draw upon this knowledge [37,38]. We posit that the kind of hierarchical visual representations that develop in our brain-inspired models, which encode not only visual features at every spatial scale but also the binding relations between these features, are necessary to enable the brain to learn a sufficiently rich model of causal relations in the world for guiding decision-making and behaviour.

An extraordinary aspect of the hierarchical feature-binding hypothesis of Eguchi *et al.* [9] is that this theory proposes a key functional role for axonal transmission delays, which theoretical neuroscientists and engineers may have previously considered to be merely sources of noise or processing delay in the primate visual system. Instead, these axonal delays are essential to the emergence of polychronization and feature-binding representations. It is therefore highly interesting to note that other simulation studies have found that axonal transmission delays may play an important functional role in quite a different aspect of brain function, that is, path integration of allocentric spatial representations in the brain. Specifically, Walters *et al.* [39] found that incorporating axonal transmission delays into their model of the head direction system allowed the model to learn to update its internal representation of head direction using vestibular angular head velocity signals at approximately the correct speed during head rotations in the dark. Taken together, these varied simulation studies indicate that axonal transmission delays may play an important role in information processing across a variety of different brain areas and functions.

However, the simulation study carried out by Eguchi *et al.* [9] was limited by the use of a relatively impoverished set of visual stimuli used to train and test the network as shown in figure 11. In particular, these authors did not test the firing responses of three-neuron binding circuits that emerged in their model on a large set of more realistic visual stimuli translating across different retinal locations. Nor did they present multiple stimuli at the same time to the network during testing, which is a further important test of feature binding as discussed by [2] and illustrated in figure 1. In such richer visual test environments, sometimes the low-level feature neuron 1 may fire without stimulating the high-level feature neuron 2 because the lower-level feature is part of a different visual object, or the high-level feature neuron 2 (with a larger receptive field) may fire without the low-level feature neuron 1 (with a smaller receptive field) being activated because the visual object is presented at a different retinal location. These kinds of more realistic simulation are needed to enable a proper test of whether such binding neurons consistently fire if and only if the low-level feature neuron 1 is participating in firing the high-level feature neuron 2. Hence this remains an important property to test for in future simulation studies with more ecologically realistic visual test scenes containing multiple objects that undergo natural transformations such as changes in retinal location, orientation or scale.

Furthermore, the role of population-wide oscillations in the coding of information is left unaddressed in this paper. Population oscillations may emerge naturally in the cortex through interactions between populations of excitatory and inhibitory neurons. Moreover, the literature indicates important functional roles for population oscillations [14] within cortical neural networks. In particular, there is experimental evidence that spatio-temporal patterns of spiking activity may occur in fixed temporal relationships to underlying population oscillations, where the timings of spikes relative to the population oscillation carry stimulus information [13]. In this case, the PNGs may sit on top of, and in fact be organized by, the underlying population oscillation. In future simulation work, we will investigate the interaction between population oscillations and both precise input spike timing and emergent polychronization.

The simulation results discussed in this paper show how representations of visual features at every spatial scale, as well as the hierarchical binding relations between these features, may develop through the emergent polychronization within biological spiking neural networks and be projected up to the higher network layers for readout by later behavioural brain systems. These theoretical findings, which are supported by neurophysiology studies such as Abeles *et al.* [23], Prut *et al.* [24] and Rainer *et al.* [18], are consistent with the rich hierarchical phenomenology of primate vision as described by Duncan & Humphreys [4] in §1. We claim that such a semantically rich hierarchical visuospatial representation is essential to the ability of the brain to make sense of its sensory world and behave intelligently within it. Understanding this ability of biological vision is therefore a key step towards the development of machines that can also perceive and understand their environment and behave flexibly within it—i.e. what is commonly referred to as artificial general intelligence.

## Data accessibility

Data from the novel simulations presented in this paper are available at: https://github.com/jamesisbister/ANewApproachToSolvingTheFeatureBindingProblemInPrimateVisionNovelData. Simulations were made using the Spike CUDA/C++ Neural Network Simulator, available at: https://github.com/OFTNAI/Spike. Simulations from the original study used the following executable: https://github.com/OFTNAI/Spike/blob/master/Experiments/ConductanceExperiment1.cpp.
